# Integrative phenotypic and transcriptomic validation of an alveolar-like macrophage model reveals early host–pathogen dynamics during *Aspergillus fumigatus* infection

**DOI:** 10.3389/fmicb.2026.1922948

**Published:** 2026-10-08

**Authors:** Jeany Söhnlein, Sascha Schäuble, Juan Prada Salcedo, Zahraa Abboud, Dalia Sheta, Kerstin Hünniger-Ast, Michelle Seif, Hermann Einsele, Thomas Dandekar, Gianni Panagiotou, Andreas Beilhack, Juergen Loeffler

**Affiliations:** 1 Department of Internal Medicine II, University Hospital Würzburg, Würzburg, Germany; 2 Department of Microbiome Dynamics, Leibniz Institute for Natural Product Research and Infection Biology, Hans-Knöll-Institute (HKI), Jena, Germany; 3 Chair of bioinformatics, Julius-Maximilian University Würzburg (JMU), Würzburg, Germany; 4 Department of Fungal Septomics, Leibniz Institute for Natural Product Research and Infection Biology, Hans-Knöll-Institute (HKI), Jena, Germany; 5 Cluster of Excellence Balance of the Microverse, Friedrich-Schiller University Jena, Jena, Germany

**Keywords:** alveolar macrophages, *Aspergillus fumigatus*, dual RNA-sequencing, fungal-host interaction, host-pathogen analysis, immune evasion

## Abstract

Primary alveolar macrophages (pAMs) are essential for the rapid clearance of conidia and maintenance of pulmonary homeostasis. However, *Aspergillus fumigatus* remains the leading cause of invasive pulmonary aspergillosis in immunocompromised patients, and the mechanisms governing fungal clearance versus invasion remain poorly understood. Although, pAMs can be isolated from human donors, their broader application in *in vitro* infection studies is limited by their low availability and technical challenges with their maintenance in culture. In this study, we successfully adapted a previously established monocyte-derived alveolar-like macrophage (ALM) model to investigate early host-pathogen interactions upon *A. fumigatus* challenge. Given the requirement of GM-CSF for maintaining alveolar macrophage identity and function, we included GM-CSF differentiated macrophages (GM-M), as a widely employed reference model. Primary alveolar macrophages (pAM), isolated from human lung biopsies were utilized to validate the physiological relevance of the ALM model. Combined phenotypic, transcriptomic and functional analyses demonstrated that ALMs closely resemble pAMs under both steady-state and infection conditions across multiple time points and fungal burdens. Notably, fungal dual RNA-sequencing revealed a significant upregulation of fungal virulence-associated factors during interaction with ALM, which was not observed in our GM-M co-cultures. Collectively, these findings support the use of ALMs as a robust, experimentally accessible and physiologically relevant *in vitro* model for investigating early *A. fumigatus* infection, providing new insights into host-pathogen dynamics at the alveolar interface.

## 1 Introduction

*Aspergillus fumigatus,* a saprotrophic mold commonly found in soil and decaying organic matter, produces 2–3 μm small airborne conidia. Its thermotolerance, broad pH adaptability, and hydrophobic conidial surface contribute to its success as an opportunistic airborne pathogen. Upon inhalation, conidia can reach the lung alveoli, where they embed within the surfactant layer of the alveolar epithelium and initiate germination within hours (Ochs et al., 2020; van de Veerdonk et al., 2017). In immunocompromised individuals, this can lead to invasive pulmonary aspergillosis (IPA), a life-threatening disease particularly dangerous for patients with neutropenia, undergoing chemotherapy or transplantation, or receiving prolonged corticosteroid therapy (Corre et al., 2010; Elkhapery et al., 2025).

Airway epithelial cells and primary alveolar macrophages (pAMs) constitute the first line of defense against *A. fumigatus*. These cells employ multiple antifungal mechanisms, including conidial phagocytosis, production of cytokines and antimicrobial peptides, and generation of reactive oxygen species (Osherov, 2012). As the dominant immune cell population in the alveolar niche under homeostatic conditions, pAMs act as key regulators of pulmonary immunity, with their phenotype and function shaped by the local tissue environment. Upon infection, they coordinate early immune responses by promoting leukocyte recruitment and directly contributing to pathogen clearance (Gosselin et al., 2014; Malainou et al., 2023; Pahari et al., 2023).

Although pAMs play a central role in the early immune response against *A. fumigatus* (Margalit and Kavanagh, 2015), underlying mechanisms remain incompletely understood. Experimental approaches have included *in vitro* and *in vivo* infection models as well as the use of isolated pAMs (Rosowski et al., 2018; Bhatia et al., 2011; Wang et al., 2022; Amich et al., 2020; Kalleda et al., 2016; Hellmann et al., 2017). However, these strategies face major limitations. pAMs rapidly lose their tissue-specific phenotype during *in vitro* culture, and their isolation is constrained by low yield, donor variability, and limited amenability to genetic manipulation (Pahari et al., 2023; Papp et al., 2018; Malainou et al., 2023). To address these challenges, recent work has established a monocyte-derived alveolar-like macrophage model (ALM) that recapitulates key features of pAMs while offering improved scalability and experimental accessibility. Although the ALM model has previously been validated in bacterial and viral infection settings, its applicability to fungal pathogens has not been investigated (Pahari et al., 2023; Pahari et al., 2024).

Here, we adapted this previously described ALM model to study host-pathogen interactions during *A. fumigatus* infection and systematically compared it to biopsy isolated human pAMs and GM-CSF differentiated macrophages (GM-Ms) derived from the same donor as ALMs. GM-Ms were included as a widely used reference model, as GM-CSF is critical for pAM development and function in the lung environment (Malainou et al., 2023; Pahari et al., 2023). Using combined phenotypic, transcriptomic and functional analyses, we demonstrate that ALMs closely resemble pAMs both under steady-state and infectious conditions, independent of fungal burden and infection time. Hence, we demonstrate that ALMs are suitable to investigate early host-pathogen interactions in *A. fumigatus* infection.

## 2 Materials and methods

### 2.1 Cell culture

CD14^+^ monocytes were isolated from peripheral blood mononuclear cells (PBMCs) as previously described (Seelbinder et al., 2020; Heilig et al., 2025). Briefly, PBMCs were obtained from healthy adults using leukoreduction chambers. Mononuclear cells were isolated by density-gradient centrifugation using Histopaque (density 1.077 g/mL; Sigma) and subsequently quantified using a Vi-Cell XR counter (Beckman Coulter). Employing a positive selection with human CD14 microbeads (Miltenyi Biotec) from PBMCs, CD14^+^ monocytes were isolated, according to manufacturer’s protocol. Differentiation into GM-Ms was achieved by supplementing Macrophage Serum-free medium (M-SFM) (Gibco) with three doses of 20 ng/mL GM-CSF (Miltenyi), while ALMs were generated by supplementing M-SFM with three doses of 100 μg/mL Curosurf (Chiesi), 10 ng/mL GM-CSF, 5 ng/mL IL-10 (Miltenyi) and 5 ng/mL TGFβ (Miltenyi) on day 0, 2, and 4. Macrophages were harvested on day 6 with accutase (Sigma) for 20 min at 37 °C, 5% CO_2_ and a cell scraper and used for further experiments.

For pAM isolation and cultivation from fresh human lung biopsies provided by the University Hospital Wuerzburg, previously published protocols were adapted (Davies and Gordon, 2005; Pahari et al., 2023). Concisely, lung tissue was dissected with scissors and pAMs were washed out with PBS (Sigma) and filtered through a 70 μm strainer. Lysis of erythrocytes was achieved with erythrocyte lysis buffer (QIAGEN) for 5 min at room temperature (RT). Cells were washed once with RPMI 1640 (Gibco) + 10% FBS + 120 μg/mL gentamicin, resuspended and seeded overnight in a 6-well plate. Afterwards, adherent cells were washed three times with PBS before harvesting with accutase and a cell scraper, centrifuged and resuspended in RPMI 1640 + 10% FBS + 120 μg/mL gentamicin for subsequent experiments. Furthermore, pAMs were experimentally evaluated within 48 h of tissue removal. Lung samples from heavy smokers were excluded. Use and processing of human blood specimen (#302/12) and pAMs (#34/22) from tissue was approved by the Ethics Committee of the University of Wuerzburg and informed consent of lung biopsy donors was obtained from each patient before surgery. To further standardize the infection environment, all Mφ populations were reseeded in RPMI 1640 + 10% FBS + 120 μg/mL gentamicin before infection. Additionally, to exclude distortion of the comparative results in functional analyses due to varying proliferation kinetics, we strictly normalized the post-harvest living cell numbers of pAM, ALM, and GM-M reseeded in each well to the exact same cell number.

### 2.2 *Aspergillus fumigatus* culture conditions

To provide optimal growth conditions, *A. fumigatus* ATCC46645, CEA17 ∆*akuB KU80*, ∆*aspf1*, and ∆*aspf2* were cultured on malt agar plates (20 g/L malt extract, 2 g/L yeast extract, 10 g/L glucose, 0.25 g/L NH_4_Cl, 0.25 g/L K_2_HPO_4_, 20 g/L agar, pH 6.0) as previously described (Krespach et al., 2023) at 37 °C for 4 days in the dark. Malt agar media was chosen due to its frequent recommendation and provision of optimal growth conditions for *A. fumigatus* (Viegas et al., 2021). Medium was supplemented with 0.1 μg/mL pyrithiamine (Sigma) for *A. fumigatus* ∆*aspf1*, and ∆*aspf2* after autoclaving. To retain stable reporter expression, *A. fumigatus* DsRed^+^ or Af293 were grown on *Aspergillus* minimal media (Scott et al., 1982; Brakhage and Van den Brulle, 1995) (6 g/L NaNO_3_, 1.52 g/L KH_2_PO_4_, 0.52 g/L KCl, 15 g/L agar, 1x Trace elements stock solution, 2.5 mL 20% MgSO_4_ x 7H_2_O stock solution, 20 mL 50% glucose stock solution, pH 6.5) and incubated at 37 °C for 7 days in the dark as described in the original protocol (Brunel et al., 2017). The medium was supplemented with 100 μg/mL hygromycin (Sigma) for DsRed^+^ conidia after autoclaving. Fungal conidia of all strains were harvested by scraping into 10 mL sterile ddH_2_O and filtered through a 40 μm cell strainer. Conidia were counted using a Neubauer hemocytometer, kept at 4 °C and used within 14 days. Detailed steps for media preparation can be found in the Supplementary Figures and Methods file.

To assess the influence of Asp f1 and Asp f2 deficiency on the germination of *A. fumigatus,* 1×10^5^ conidia of *A. fumigatus* CEA17, *∆aspf1* and *∆aspf2* were inoculated in RPMI 1640 and incubated at 37 °C, 5% CO_2_ for 4 h-9 h. At each time point, 10 pictures were taken using a Zeiss Primovert Microscope using a 40x objective lens. During data acquisition, clumps of conidia/hyphae were not considered (Jia et al., 2025).

### 2.3 Phenotypic evaluation of pAM, ALM and GM-M

For immunofluorescence analysis, pAM, ALM or GM-M were blocked with 5% BSA/PBS for 10 min at 4 °C and stained with anti-CD206 AF488 (1:20; Biolegend) and anti-CD11c AF647 (1:10; Biolegend) at 4 °C for 20 min. Cells were washed three times with PBS, resuspended in RPMI 1640 + 10% FBS + 120 μg/mL gentamicin and 1×10^5^ macrophages were seeded into μ-Slide VI 0.4 chambers (ibidi). After overnight incubation at 37 °C, 5% CO_2_, cells were stained with Hoechst (1:2,000; Thermo Fisher) in PBS for 10 min at RT followed by three times washing with PBS. Supernatant was removed and exchanged to RPMI 1640 + 10% FBS + 120 μg/mL gentamicin and imaged using a Zeiss LSM780 confocal microscope (Zeiss, Germany).

For flow cytometry, 1×10^5^ cells were blocked with human Fc receptor blocking reagent (Miltenyi), according to manufacturer’s protocol, at 4 °C for 10 min and stained with fixable Viability Dye eFluor™ 780 (1:2,000; Invitrogen™), anti-HLA-DR-DP-DQ VioB515 (1:100; Miltenyi), anti-CD206 PECy7 (1:100; Biolegend), anti-CD11b APC (1:50; Miltenyi) or the respective isotype control at 4 °C for 20 min. Inside staining was performed using the Inside Stain kit (Miltenyi) according to manufacturer’s protocol and anti-CD68 BV785 (1:20; Biolegend). Samples were analyzed on a CytoFlex Flow Cytometer (Beckman Coulter) and data was processed using Kaluza V2.2.1 software (Beckman Coulter). Furthermore, CD11c and CD206 double positivity was used to evaluate the purity of isolated pAMs (Supplementary Figure S1B) and gated after ALMs due to the high autofluorescent background in the pAM background controls. This yielded an average pAM purity of ~91%.

### 2.4 Macrophage infection assays

pAM, ALM and GM-M were infected with resting *A. fumigatus* ATC46645 conidia at a multiplicity of infection (MOI) of 1 for 6 h. Alternatively, cells were infected at MOI 5 for 9 h using strains *A. fumigatus* ATCC46645 or CEA17, ∆*aspf1*, or ∆*aspf2*. For dual RNA-sequencing, ALM and GM-M were infected with *A. fumigatus* ATCC46645 at MOI 5 for 6 and 9 h. Infection was synchronized by centrifugation at 200xg, 5 min before incubation. Following infection, cells were harvested by scraping and centrifugation at 300xg, 5 min. Supernatants or cell pellets in RNAprotect Cell Reagent (QIAGEN) were stored at −80 °C until further processing.

### 2.5 Phagocytosis and FLARE infection assay

Surface labeling of DsRed^+^ conidia was conducted as previously described (Brunel et al., 2017; Jhingran et al., 2012). FLARE conidia were stored in PBS at 4 °C for a maximum of 2 weeks after harvesting. ALM and GM-M were infected with *A. fumigatus* FLARE at a MOI of 0.5, 1, or 3 or with Af293 MOI 3 for isotype control and centrifuged at 200xg, 5 min to initiate proper infection, followed by incubation for 2 or 6 h at 37 °C, 5% CO_2_. FLARE infected cells were counterstained with calcofluor white (CFW) in RPMI 1640 + 10% FBS + 120 μg/mL gentamicin (1:40; Sigma) for 10 min at 37 °C, 5% CO_2_, to distinguish between extracellular and phagocytosed conidia. Macrophages were blocked with Fc receptor blocking solution and stained with fixable Viability Dye eFluor™ 780 (1:2,000), anti-CD206 BV785 (1:20; Biolegend) and anti-CD11c PEV770 (1:50; Miltenyi). Phagocytosis and killing efficiency were analyzed on a CytoFlex Flow Cytometer by measuring 10,000 cells in the single cells gate and evaluated using Kaluza V2.2.1 software. For representative pictures, ALM were blocked with 5% BSA/PBS, stained with anti-CD206 AF488 (1:20; Biolegend) and seeded overnight into μ-Slide VI 0.4 chambers (ibidi). Cells were infected using FLARE conidia at MOI 3 and incubated for 6 h before counterstaining with CFW. Microscopy pictures were obtained employing a Zeiss CLSM780 confocal microscope.

To quantify the phagocytosis of *A. fumigatus* CEA17, ∆*aspf1*, or ∆*aspf2* conidia, ALMs were infected with a MOI 5 of each fungal strain for 2 h. Infected cells were treated as described above and phagocytic efficiency was quantified on a CytoFlex Flow Cytometer (Beckman Coulter) and evaluated using Kaluza V2.2.1 software.

To evaluate the surface expression of potential phagocytic mediators TLR2 and Dectin-1 on the Mφ models, ALMs and GM-Ms were blocked with human FcR blocking solution (Miltenyi) according to manufacturer’s protocol for 10 min at 4 °C before staining with fixable Viability Dye eFluor™ 780 (1:2,000; Invitrogen™), anti-Dectin-1 PEVio770 (1:20), anti-TLR2 APC (1:25), anti-CD11c VioBlue, (1:50) and anti-CD206 BV785 (1:20) for 20 min at 4 °C. CD11c and CD206 were used as Mφ markers to ensure ALM and GM-M phenotype (Supplementary Figure S1B). Samples were analyzed on a CytoFlex Flow Cytometer (Beckman Coulter) and data was processed using Kaluza V2.2.a software (Beckman Coulter).

### 2.6 RNA isolation and dual bulk transcriptomic profiling

RNA was isolated using the RiboPure™ RNA-purification kit yeast (Invitrogen) including DNAse treatment according to manufacturer’s protocol. Isolated RNA was measured on a NanoQuant Infinite M200 Pro plate reader (Tecan). RNA quality was checked using a 5,200 Fragment Analyzer with the DNF-471-33-SS Total RNA 15 nt kit (Agilent Technologies). DNAse-treated RNA was depleted of ribosomal RNA molecules using RiboCop for Human/Mouse/Rat V2 (Cat. No. 144) rRNA depletion kit (Lexogen). The ribo-depleted RNA samples were first fragmented using ultrasound (4 pulses of 30s at 4 °C). Then, an oligonucleotide adapter was ligated to the 3′ end of the RNA molecules. First-strand cDNA synthesis was performed using M-MLV reverse transcriptase with the 3′ adapter as primer. After purification, the 5’ Illumina TruSeq sequencing adapter was ligated to the 3′ end of the antisense cDNA. The resulting cDNA was PCR-amplified using a high-fidelity DNA polymerase and the barcoded TruSeq-libraries were pooled in approximately equimolar amounts. The size distribution of the barcoded DNA libraries was estimated to be ~400 bp by electrophoresis on 5,200 Fragment Analyzer with the DNF-474-33-HS NGS Fragment 1-6000 bp kit (Agilent Technologies). Sequencing of pooled libraries, spiked with PhiX control library, was performed at 50 million reads per sample in single-ended mode with 100 cycles on the NovaSeq 6,000 platform (Illumina). Demultiplexed FASTQ filed were generated with bcl-convert v4.2.4 (Illumina).

### 2.7 Dual RNA-sequencing processing and pathway analysis

Raw sequencing reads including quality control and gene abundance estimation were processed using the GEO2RNAseq pipeline (v0.9.12) (Seelbinder et al., 2020) implemented in *R* (v3.5.1). Read quality was assessed before and after trimming using FastQC (v0.11.8). Low-quality bases and adapter sequences were removed with Trimmomatic (v0.36) and ribosomal RNA reads were filtered using SortMeRNA (v2.1) with a combined database containing all rRNA references provided by SortMeRNA. To enable simultaneous host-pathogen analysis, a combined reference genome was generated by merging the *Homo sapiens* reference genome (GRCh38, version 89) with the *A. fumigatus* Af293 reference genome (version s03-m05-r05) in FASTA format. Corresponding exon annotations were extracted and combined into a unified annotation file. The merged reference genome was indexed with exon information using HiSat2 (v2.1.0) (in single-end mode) and gene abundance estimation was performed with featureCounts (v1.28.0) using default parameters. MultiQC (v1.7) was used to summarize the output of FastQC, Trimmomatic, HiSat, featureCounts and SAMtools. The read count matrix with gene abundances for both species without and with median-of-ratios normalization (MRN) (Anders and Huber, 2010) were extracted. Differential gene expression per organism was analyzed using GEO2RNaseq. Pairwise tests were performed using four statistical tools (DESeq v1.30.0, DESeq2 v1.18.1, limma voom v3.34.6 and edgeR v3.20.7) to report *p* values and multiple testing corrected *p* values using the false-discovery rate method q = FDR(p) for each tool. Gene expression differences were considered significant if they were reported significant by all four tools (*p* ≤ 0.05) and a minimum fold change of log_2_(FC_MRN_) ≥ 1. Gene Ontology (GO) enrichment analysis was performed in *R* using the GSEGO function, with genes ranked according to $\log_{2}$ fold change values derived from DESeq2 differential expression analysis. For human gene annotation, the org. Hs.eg.db package from AnnotationDbi was used, while *A. fumigatus* Af293 annotations were obtained from a publicly available resource (Pardeshi, 2021). Enriched GO terms were visualized using dot plots.

### 2.8 Host-pathogen protein–protein interaction analysis

To prioritize biological processes relevant to host–pathogen interactions, enriched GO terms were filtered using a predefined curated list of interaction-associated terms provided in the Supplementary Table S1. This filtering included extracellular and cell wall–associated terms within the Cellular Component (CC) ontology as well as interaction-relevant Molecular Function (MF) categories. Differentially expressed genes (DEGs) were retained when annotated to selected GO terms in both CC and MF categories, ensuring consistency between subcellular localization and molecular function. Subsequently, we filtered for predicted protein features associated with host–pathogen interactions using established computational tools, including NetGPI-1.1 (GPI-anchor prediction) (Gíslason et al., 2021; Gíslason, 2021), SignalP 6.0 (signal peptide prediction) (Teufel et al., 2022), TargetP 2.0 (secretory pathway targeting) (Almagro Armenteros et al., 2019), DeepTMHMM 1.0 (transmembrane domain prediction) (Hallgren et al., 2022), and DeepLoc 2.1 (subcellular localization and membrane protein classification) (Ødum et al., 2024). Proteins were retained if they contained at least one interaction-associated feature, including a GPI anchor, signaling peptide, predicted secretion signal, appropriate subcellular localization, or one or more transmembrane domains. To further identify potential virulence-associated factors, *A. fumigatus* proteins were queried against the PHI-base database (Urban et al., 2025). *A. fumigatus* candidate genes were subsequently prioritized using a composite scoring approach integrating functional annotation and predicted protein features to enrich for proteins potentially involved in direct host–pathogen interactions. A pathogen relevance score was assigned to *A. fumigatus* proteins based on functional annotations and protein descriptions using a custom *R* function. Briefly, proteins were classified into five hierarchical tiers reflecting their potential involvement in host–pathogen interactions, with only the highest-ranking matching category retained per protein. Tier 1 (score = 10) included direct pathogenicity factors such as allergens, toxins, virulence-associated proteins, invasins, and adhesins, as well as annotations related to pathogenesis or immune evasion. Tier 2 (score = 8) comprised cell wall–degrading enzymes, including chitinases, glucanases, and non-ribosomal proteases/peptidases, along with corresponding GO terms. Tier 3 (score = 6) included enzymes involved in tissue degradation, such as cellulases, xylanases, and pectinases. Tier 4 (score = 4) captured proteins associated with host adaptation and stress responses, including catalases, superoxide dismutases, and heat shock proteins. Tier 5 (score = 3) included proteins predicted to be secreted or localized to the cell surface. Classification was performed by matching protein names and GO annotations (biological process, molecular function, and cellular component) using case-insensitive pattern recognition. Proteins not matching any category were assigned a score of 0 and labeled as not relevant. Finally, protein–protein interaction (PPI) networks were constructed using interaction data retrieved from the STRING database (DB) version 12.0. We used all possible interactions and a confidence threshold of 0.4.

### 2.9 cDNA synthesis and quantitative reverse transcription PCR

To validate the fungal transcriptomic profile observed in the dual RNA-sequencing analysis, total RNA was isolated with the RiboPure™ RNA-purification kit yeast (Invitrogen) including DNAse treatment according to manufacturer’s protocol. The RNA was converted into cDNA using the High-Capacity cDNA Reverse Transcription Kit (Applied Biosystems™). qRT-PCR reactions were performed in duplicates employing 10 nM gene-specific primers and iTaq™ Universal SYBR® Green Supermix (BioRad Laboratories) on a StepOnePlus™ Real-Time PCR System (Applied Biosystems™). Relative gene expression was calculated using the ∆∆Ct method relative to the house-keeping gene *act1*. Primers are listed in the Supplementary Table S2. Primers for *act1*, *gliZ*, *cat1*, and *cipC* were selected from published data (Canela et al., 2017; Seelbinder et al., 2020; Sueiro-Olivares et al., 2015).

### 2.10 Cytokine analysis

Custom ProcartaPlex (Thermo Fisher) or custom LEGENDplex (Biolegend) assay kits were used for multiplexed quantification of cytokine concentrations in culture supernatants according to the manufacturer’s protocol. ProcartaPlex data acquisition and analysis were performed using a Bio-Plex 200 Luminex reader in combination with Bio-Plex Manager Software version 6.2 (Bio-Rad). LEGENDplex data acquisition and analysis was performed on a CytoFlex Flow Cytometer (Beckman Coulter) according to manufacturer’s protocol. CCL2 concentrations measured out-of-range high were capped at the top standard of 10,000 pg./mL. Furthermore, due to the high occurrence of IL-8 concentrations, which were out-of-range high, a single-plex ELISA was performed for pAMs, ALMs and GM-Ms infected with *A. fumigatus* CEA17, *∆aspf1* and *∆aspf2* MOI 5 for 9 h. Single-plex ELISA assays including ELISA MAX Deluxe Set Human for TNF, IL-8, CXCL10 (Biolegend) or Human DuoSet kits for CCL3 and CCL4 (R&D Systems) were performed according to manufacturer’s protocol.

### 2.11 Colony forming unit analysis

ALMs were infected with MOI 5 of *A. fumigatus* CEA17, *∆aspf1* and *∆aspf2* for 9 h. Subsequently, cells were lysed with cold ddH_2_O. The conidia suspensions were quantitatively cultured by serial dilution, plated on malt agar plates and incubated at 37 °C for 18 h. Afterwards, the *A. fumigatus* fungal burdens (number of CFU/mL) were determined (Xiong et al., 2023). ALMs were chosen due to their pAM resembling cytokine profile during infection with each of the three tested strains.

### 2.12 Statistical analysis

GraphPad Prism version 10, Kaluza version 2.2.1, and *R* version 3.5.1 were used for data compilation, analysis and visualization. Depending on the data format, significance testing was performed using paired *t*-test, Kruskal-Wallis Test with Dunn’s multiple comparison, Ordinary One-Way ANOVA with Sidák’s or Tukey’s Multiple Comparison, RM-ANOVA with Dunnet’s Multiple Comparison, or Two-Way ANOVA with Tukey’s Multiple Comparison. The specific significance tests are specified in the figure legends. *p*-values < 0.05 were considered significant.

## 3 Results

### 3.1 Alveolar-like macrophages resemble primary alveolar macrophages under steady-state and *Aspergillus fumigatus* infection conditions

To investigate early alveolar macrophage (Mφ) responses to *A. fumigatus*, we first assessed whether our differentiation protocol generated ALMs that exhibit key features of pAMs under steady-state and infection (Figure 1A). Morphological analysis using immunofluorescence staining of established pAM markers (Supplementary Figure S1A) (Bain and MacDonald, 2022) revealed that both pAMs and ALMs displayed a predominantly round morphology, consistent with previous reports (Pahari et al., 2023), whereas GM-Ms appeared flatter and more elongated (Figure 1B). pAMs exhibited a larger cell size compared to ALMs, consistent with flow cytometry measurements (Figure 1C). Moreover, pAMs showed higher autofluorescence, a characteristic feature of tissue-resident cells, whereas ALMs and GM-Ms exhibited lower autofluorescence (Figure 1D) likely reflecting their monocyte-derived origin (Knab et al., 2025). To further characterize Mφ identity, we analyzed the expression of canonical pAM markers, including signature markers CD206 and CD68 (Supplementary Figure S1B) (Sun et al., 2026; Bain and MacDonald, 2022). Flow cytometric analysis revealed gradual differences in marker expression across the three Mφ populations, particularly for HLA II and CD11b (Figure 1D). Quantitative analysis of mean fluorescence identity (MFI) demonstrated that ALMs expressed HLA-DR-DP-DQ and CD68 at levels comparable to pAMs (Figure 1E). For CD206 and CD11b, ALMs showed intermediate expression, more closely resembling pAMs than GM-Ms, which exhibit significant differences to pAMs.

**Figure 1:**
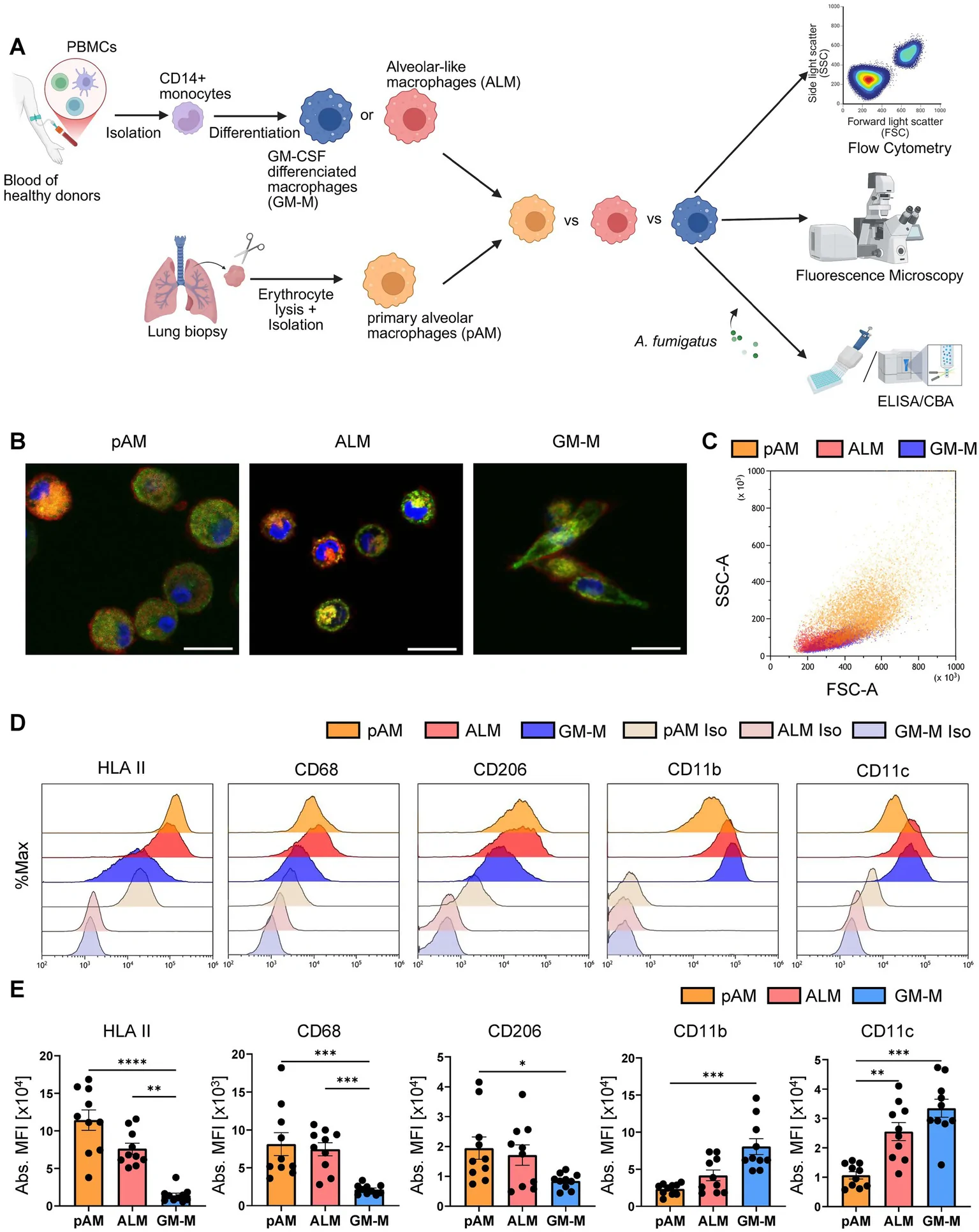
ALM resemble pAM in steady-state. **(A)** Schematic visualization of assays used to compare pAMs to ALMs and GM-Ms. Created with Biorender.com. **(B)** Phenotypic evaluation of pAM, ALM and GM-M using fluorescence microscopy. The pictures were taken on a ZEISS CLSM780 with a 40x objective and cropped for better visualization. Scale bar = 20 µm. **(C)** pAM, ALM and GM-M show differences in size and granularity in flow cytometry visualized by the SSC-A and FSC-A. **(D)** Flow cytometry analysis of pAM markers selected from Bain and MacDonald (2022). Cells were stained with indicated antibodies or the corresponding isotype controls. Representative histograms visualize mean fluorescence intensity (MFI) of individual macrophage population markers as well as background controls. **(E)** Quantification of the MFI measured by flow cytometry analysis. *n* = 10 +/− SEM, Kruskal-Wallis-test, Dunn’s Multiple Comparison; * *p* < 0.05, ** *p* < 0.01, *** *p* < 0.001, **** *p* < 0.0001.

Having established that ALMs closely resemble pAMs under steady-state conditions, we next evaluated functional responses to *A. fumigatus* infection by measuring cytokine secretion (Figure 2A). Following *A. fumigatus* ATCC46645 infection at MOI 1 for 6 h, both pAMs and ALMs exhibited robust induction of cytokines (Figure 2B; Supplementary Figure S2A) in contrast to GM-Ms. This was particularly evident for TNF, CCL3, CCL4, IL-1b, and IL-8. Additionally, ALMs showed significant induction of CCL5, CXCL9, CXCL10, and CXCL11 relative to uninfected controls, whereas pAMs displayed higher baseline secretion of these cytokines. GM-Ms exhibited limited responsiveness, with significant induction observed solely for CXCL9, alongside significantly elevated baseline levels of IL-1b, IL-8, and CCL5 compared to ALM. Increase of fungal burden (MOI 5) and extended incubation (9 h) displayed maintenance of elevated cytokine response in pAM and ALM infected cells (Figure 2C), particularly for TNF, CCL3, and CCL4. Comparison between pAMs and GM-Ms or ALMs and GM-Ms furthermore displayed significant differences in the TNF or CCL4 cytokine levels secreted by infected cells. In addition, GM-Ms failed to secrete CCL8, CCL20, IL-6, or CXCL10 while ALMs exhibited increased CXCL10 secretion, which we did not observe for pAMs due to high background secretion. No substantial differences were observed for CCL2 (Supplementary Figure S2B). Collectively, our data indicates that ALMs share key phenotypic and functional characteristics with pAMs under both steady-state and *A. fumigatus* infection conditions.

**Figure 2:**
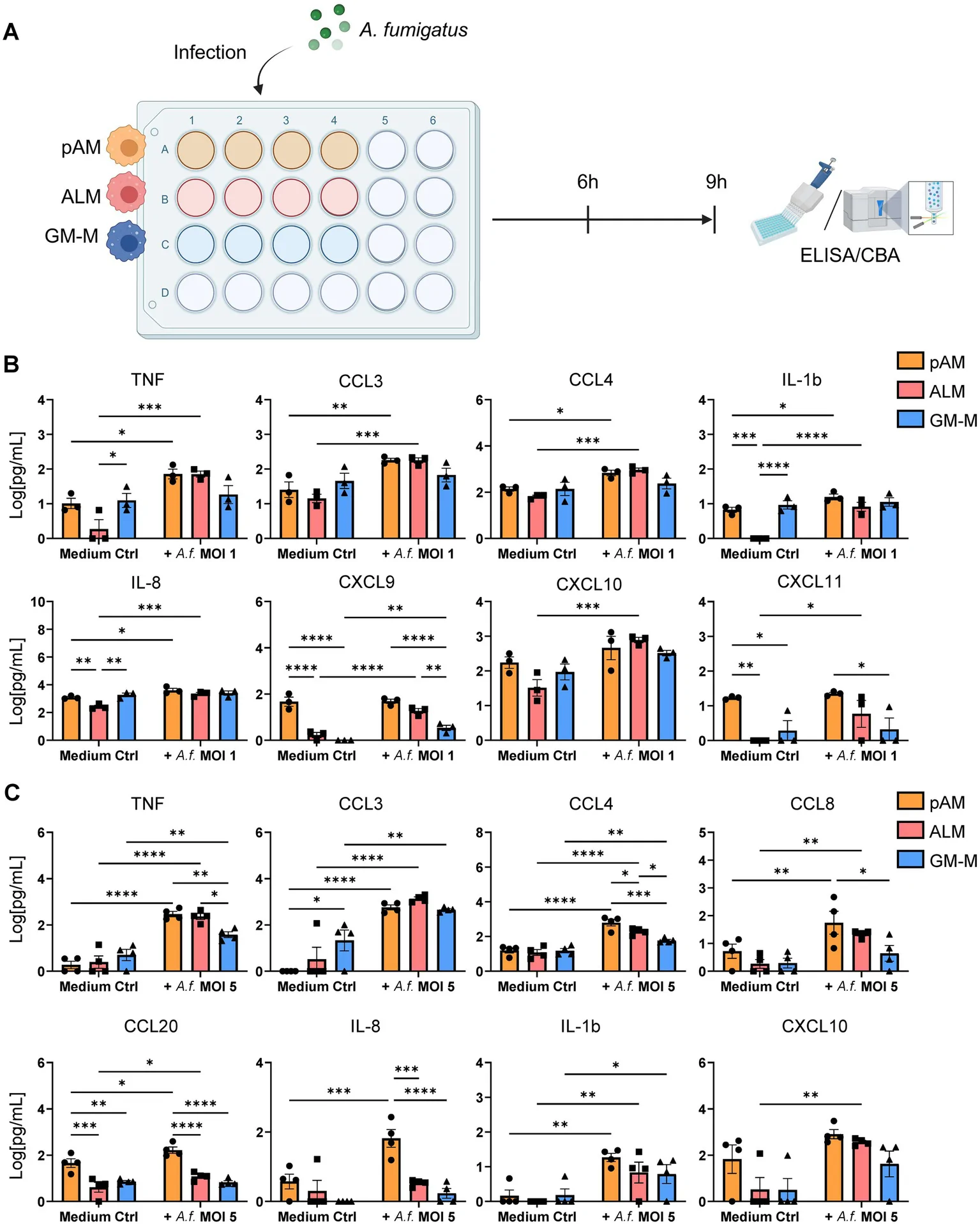
ALM resemble pAM during *Aspergillus fumigatus* infection regardless of fungal burden or infection time. **(A)** Schematic visualization of functional assays. pAM, ALM and GM-M were infected with *A. fumigatus* ATCC46645 at an MOI 1 for 6 h or at an MOI 5 for 9 h. Figure created with Biorender.com. **(B)** Cytokine secretion of pAM, ALM and GM-M after infection with *A. fumigatus* ATCC46645 MOI 1 for 6 h. Data was transformed using log(Y + 1). Cytokines were measured by employing a custom ProcartaPlex assay kit. *n* = 3 +/− SEM, Ordinary One-Way ANOVA, Tukey’s Multiple Comparison, * *p* < 0.05, ** *p* < 0.01, *** *p* < 0.001, **** *p* < 0.0001. **(C)** Evaluation of macrophage secreted cytokines upon infection with *A. fumigatus* ATCC46645 MOI 5 for 9 h. The data was transformed using log(Y + 1). *n* = 4 +/− SEM, Two-Way-ANOVA, Tukey’s Multiple Comparison, * *p* < 0.05, ** *p* < 0.01, *** *p* < 0.001, **** *p* < 0.0001.

### 3.2 ALM efficiently phagocytose *Aspergillus fumigatus* conidia

Given the observed phenotypic and functional distinctions between Mφ models, we aimed to further characterize the functional capacity of ALMs in comparison to GM-Ms. GM-Ms have been widely used to investigate host-pathogen interactions with *A. fumigatus* (Gonçalves et al., 2020; Günther et al., 2024; Brummer et al., 2001), providing a valuable reference framework for our analyses. Building on this foundation, we evaluated key Mφ effector functions, including phagocytosis and intracellular killing, central for antifungal defense (Luther et al., 2008). We employed the *A. fumigatus* fluorescent reporter strain (FLARE), which combines a DsRed signal to indicate conidial viability with a fluorescent tracer that persists after conidial death. Following phagolysosomal uptake of conidia, the DsRed signal diminishes within approximately 45 min, thereby serving as a proxy for intracellular conidial killing (Jhingran et al., 2012; Maselli et al., 2002). Representative gating strategies and microscopy images are provided in the Supplementary Figures S3A,B.

As outlined in Figure 3A, we infected ALMs and GM-Ms with FLARE-labeled conidia at MOIs ranging from 0.5 to 3 and analyzed uptake and killing at 2 h and 6 h post-infection. The time points were selected as phagocytosis in murine Mφs was proven to last up to 2 h and killing begins after 6 h of phagocytosis (Philippe et al., 2003). We distinguished extracellular from internalized conidia using CFW staining. Quantitative analysis revealed that ALMs phagocytosed conidia significantly more rapidly and efficiently than GM-Ms across all tested conditions (Figure 3B). Despite these differences in uptake kinetics, both Mφ populations exhibited comparable killing efficiencies. Within ALMs, however, killing efficiency varied depending on fungal load and increased over time. Time-course analysis further demonstrated that ALMs progressively enhanced conidial uptake, particularly at higher MOIs (Figure 3C), indicating sustained phagocytic activity under increased fungal challenge. Consistently, intracellular killing by ALMs significantly increased over time across all conditions.

**Figure 3:**
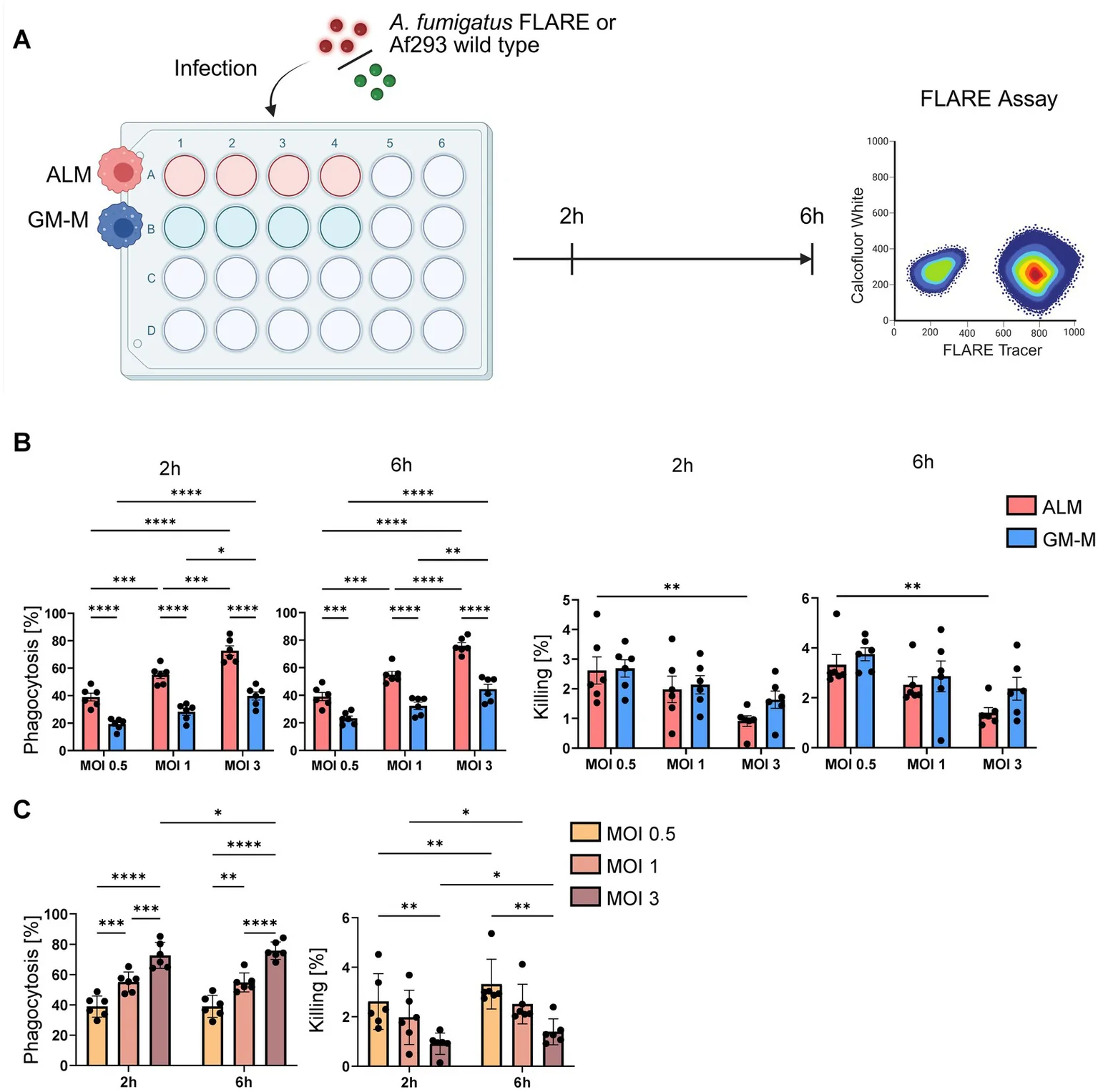
ALMs exhibit fast and efficient phagocytosis of *A. fumigatus*. **(A)** Visual representation of the FLARE infection setup. Macrophages infected with *A. fumigatus* Af293 MOI 3 were used as isotype control to distinguish between positive staining and background signals as well as killed and live conidia. Created with **(B)** Evaluation of FLARE infected ALM and GM-M. ALM and GM-M were infected with an MOI 0.5–3 for 2 h and 6 h Biorender.com respectively before counterstaining with calcofluor white (CFW). Phagocytosis evaluated the percentage of macrophages which have phagocytosed any conidia. Killing efficacy is displayed as percentage of macrophages which contain killed conidia from all macrophages which phagocytosed conidia. *n* = 6 +/− SEM, Two-Way ANOVA, Tukey’s Multiple Comparison, *p < 0.05, ** *p* < 0.01, *** *p* < 0.001, **** *p* < 0.0001. **(C)** Time dependent differences in phagocytic and killing efficacy of ALM cells. *n* = 6 +/− SEM, Two-Way ANOVA, Tukey’s Multiple Comparison, * *p* < 0.05, ** *p* < 0.01, *** *p* < 0.001, **** *p* < 0.0001.

GM-Ms also displayed time-dependent increases in conidial uptake, although at lower overall levels compared to ALMs, and showed only modest increases in killing efficiency (Supplementary Figure S3C).

The pattern-recognition receptors (PRR) Dectin-1 and TLR2 are described as two important factors of *A. fumigatus* phagocytosis (Goodridge et al., 2011; Luther et al., 2007) and might be influencing factors for the observed differences between ALMs and GM-Ms. We stained both Mφ populations for the PRRs in flow cytometry, which was used to quantify the absolute MFI (Supplementary Figure S3D). Both Dectin-1 and TLR2 were detected on the surface of ALMs and GM-Ms but significant distinctions were not observed.

In summary, these findings demonstrate that ALMs combine rapid and efficient phagocytic uptake with effective intracellular killing of *A. fumigatus* conidia, consistent with key functional properties attributed to pAMs in the lung (Luther et al., 2008).

### 3.3 ALM mount a rapid and robust pro-inflammatory transcriptional response to *Aspergillus fumigatus*

Building on the previous observations that ALMs resemble pAMs phenotypically and mechanistically, as well as rapidly phagocytose *A. fumigatus* conidia, we next sought to characterize the transcriptional response in uninfected cells and upon fungal challenge. To this end, we performed dual RNA-sequencing using ALMs and GM-Ms generated from matched donors. Both Mφ populations were infected with *A. fumigatus* conidia (MOI 5) for 6 h and 9 h, alongside uninfected controls to simultaneously assess host defense and fungal counter-defense mechanisms (Figure 4A) related to variations in the fungal morphotype. Principal component analysis (PCA) clearly separated all experimental conditions based on infection status and Mφ differentiation (Figure 4B).

**Figure 4:**
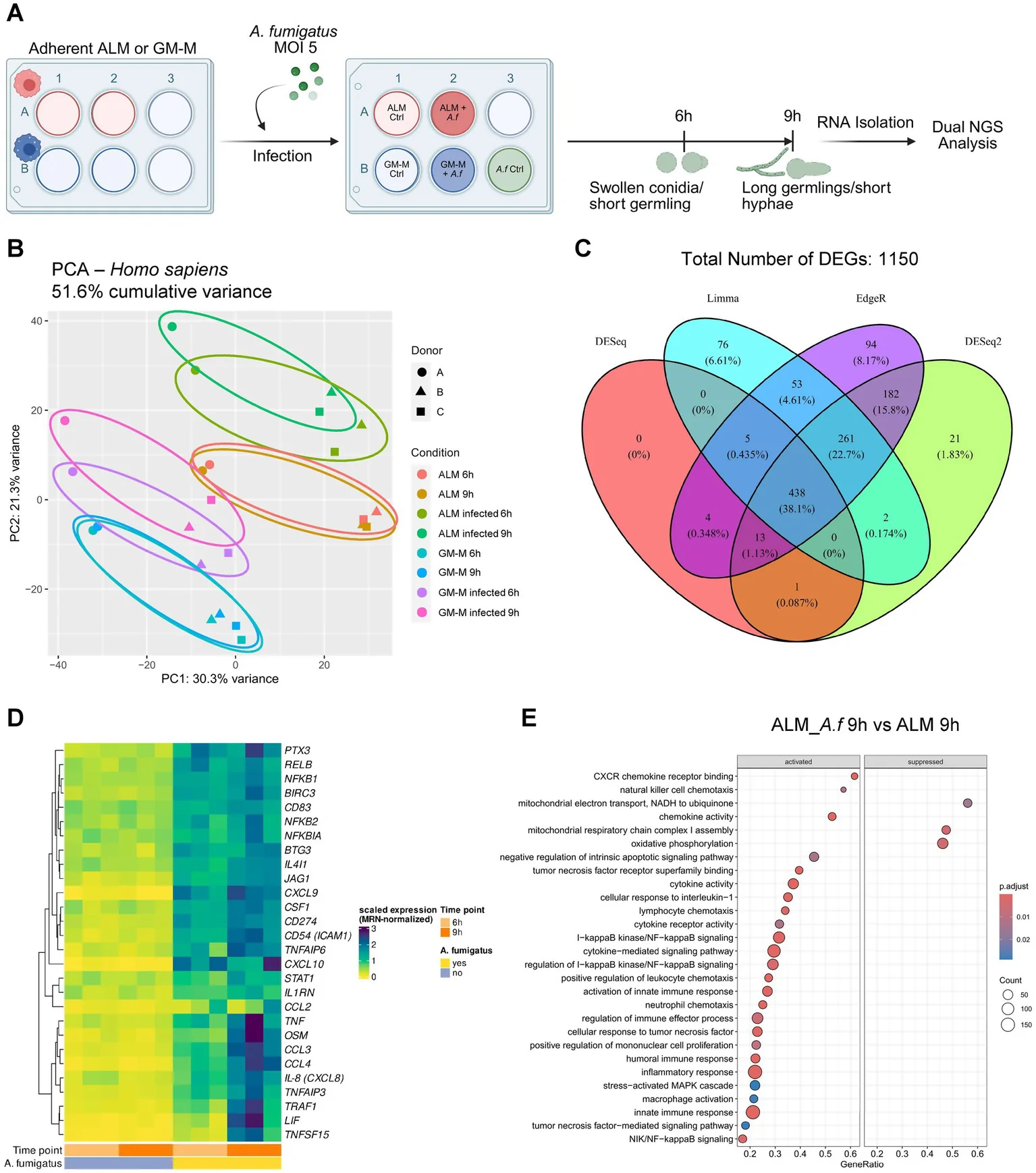
ALMs react with a fast upregulation of pro-inflammatory genes and respective pathways upon *A. fumigatus* challenge. **(A)** Representative figure to visualize the infection setup used for the dual RNA-sequencing analysis. Created with Biorender.com. **(B)** PCA plot visualizing donor (*n* = 3) and condition variances for the human transcriptome after quality control. **(C)** Venn diagram of ALMs infected for 9 h with *A. fumigatus* ATCC46645 MOI 5. **(D)** Heatmap highlighting the expression level of infection relevant genes of ALM uninfected or infected for 6 h and 9 h with *A. fumigatus* ATCC46645 MOI 5. **(E)** Pathway analysis of ALMs infected for 9 h with *A. fumigatus* ATCC46645 MOI 5. ALM cells quickly downregulate genes for oxidative phosphorylation and further upregulate pro-inflammatory pathways like NF-kB- or TNF-signaling at 9 h of infection.

We analyzed the transcriptomic profile of uninfected Mφs, screened published literature regarding pAM signature genes (Pahari et al., 2023; Kim et al., 2020; Sikkema et al., 2023; Groves et al., 2025; Aegerter et al., 2022; Deprez et al., 2020; Eddens et al., 2024) and visualized the data (Supplementary Figure S4A) according to previously published studies (Yu et al., 2017; Thomas et al., 2026). Consistent with our phenotypic and mechanistic analyses, ALMs show a markedly stronger enrichment of pAM signature genes in direct comparison with GM-Ms, strengthening the pAM resemblance. On the other hand, GM-Ms strongly express FCN1 and FN1, markers of recruited Mφs or monocytes (Aegerter et al., 2022; Li et al., 2022; Lee et al., 2024), hinting that GM-CSF-differentiated Mφs more closely resemble blood-recruited Mφs rather than pAMs. These results provide an additional quantitative validation of the physiological relevance of the ALM differentiation protocol and further demonstrate the pAM resemblance of the ALM model.

Upon infection, ALMs underwent pronounced transcriptional reprogramming at both time points, with 243 differentially expressed genes (DEGs) at 6 h (Supplementary Figure S4B) and 438 DEGs at 9 h (Figure 4C). Visualization of infection- and activation-associated genes (Margalit and Kavanagh, 2015; van de Veerdonk et al., 2017) demonstrated that ALMs maintained a homeostatic profile in uninfected conditions, whereas infection triggered a strong induction of pro-inflammatory mediators (Figure 4D). These include TNF, CCL3, CCL4, IL-8, CXCL9, and CXCL10. In addition, ALMs upregulated immune regulatory markers such as CD274 and CD83 and the adhesion molecule ICAM1. Pathway analysis further revealed that ALMs undergo metabolic reprogramming upon infection, characterized by suppression of oxidative phosphorylation at both 6 h and 9 h (Supplementary Figure S4C; Figure 4E), alongside activation of canonical inflammatory pathways, including NF-kB- and TNF-signaling, as well as pathways associated with cell recruitment (Figure 4E).

GM-Ms also responded to infection but exhibited delayed and gradual transcriptional adaptation. At 6 h post-infection, GM-Ms showed a limited response with only 16 DEGs, which increased to 283 DEGs at 9 h (Supplementary Figure S4D). Elevated baseline expression of inflammatory genes in uninfected GM-Ms indicates a pre-activated state, which may influence their response dynamics (Supplementary Figure S4E). By 9 h, GM-Ms upregulated cytokine- and chemokine-associated pathways. However, activation of NF-kB- and TNF-signaling, as well as immune cell recruitment-related pathways, remained less pronounced compared to ALMs (Supplementary Figure S4F). Like ALMs, GM-Ms exhibited suppression of oxidative phosphorylation.

We validated these transcriptional findings at the protein level using multiplex cytokine analysis of the three initial donors (Figure 5A; Supplementary Figure S5). ALMs significantly secreted TNF, CCL3, CCL4, IL-1b, and IL-6 as early as 6 h, with increase at 9 h. In contrast, GM-M displayed delayed cytokine production and lower overall secretion levels, even at 9 h post-infection. Validation in an expanded cohort of six donors (Figure 5B) confirmed these patterns, with ALMs consistently producing higher levels of TNF, CCL3, CCL4, and CXCL10 than GM-M, particularly at 9 h of fungal challenge. Collectively, these results demonstrate that ALMs mount a rapid, coordinated and robust pro-inflammatory response to *A. fumigatus,* highlighting their value as a physiologically relevant *in vitro* model for studying early host-pathogen interactions.

**Figure 5:**
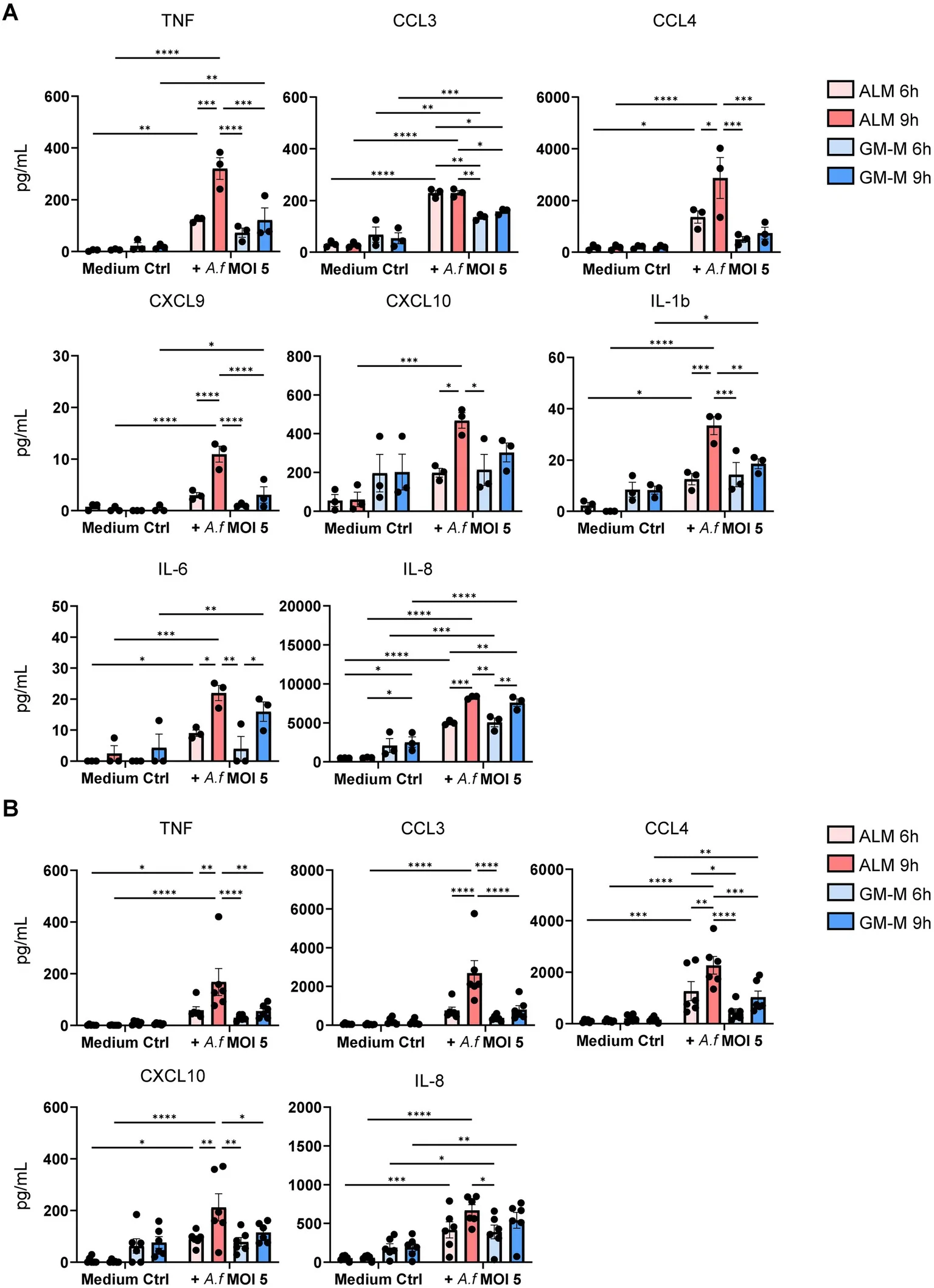
ALMs secrete significantly more cytokines than the typical blood-derived GM-Ms. **(A)** Quantification of cytokine secretion in supernatant of ALM and GM-M donors used for dual RNA-sequencing analysis. ALM and GM-M were left uninfected or infected with *A. fumigatus* ATCC46645 MOI 5 for 6 h and 9 h. The cytokines were measured employing a custom ProcartaPlex assay kit. n = 3 +/− SEM, Two-Way ANOVA, Tukey’s Multiple Comparison; * *p* < 0.05, ** *p* < 0.01, *** *p* < 0.001, **** *p* < 0.0001. **(B)** Validation of the dual RNA-sequencing dataset by singleplex ELISA including an expanded cohort. *n* = 6 +/− SEM, Two- Way ANOVA, Tukey’s multiple comparison; * *p* < 0.05, ** *p* < 0.01, *** *p* < 0.001, **** *p* < 0.0001.

### 3.4 *Aspergillus fumigatus* transcriptome analysis mounts an enhanced counter-defense response during interaction with ALMs

To complement our analysis of host responses, we next investigated fungal transcriptional adaptations during Mφ interaction (Figure 6A). PCA revealed clear clustering of fungal samples according to Mφ type and infection time point, indicating distinct transcriptional programs under each condition (Figure 6B). During interaction with ALMs, *A. fumigatus* underwent extensive transcriptional reprogramming, particularly at 9 h, with 2,325 DEGs (Figure 6C). Compared to unchallenged controls, the fungus strongly upregulated genes associated to stress adaptation and immune evasion, including *cat1* (Afu3g02270) (Abad et al., 2010; Keizer et al., 2022) or key components of the gliotoxin biosynthetic pathway (*gliZ*/Afu6g09630, *gliA*/Afu6g09710, *gliG*/Afu6g09690) (Knowles et al., 2020). Additionally, *A. fumigatus* induced allergen-encoding genes such as *aspf1* (Afu5g02330) and *aspf2* (Afu4g09580), with *aspf1* showing significant induction during interaction with ALMs (Figure 6D). Pathway analysis revealed a dynamic transcriptional response characterized by suppression of growth-associated processes, including ergosterol biosynthesis and nuclear division (Alcazar-Fuoli et al., 2008), alongside activation of transcriptional regulation pathways at 6 h (Figure 6E, left panel). At 9 h, the fungal response shifted toward activation of virulence-associated programs, including gliotoxin production, secondary metabolite biosynthesis, and IgE binding pathways (Figure 6E, right panel), indicating an intensified adaptive fungal response to ALM-mediated immune pressure.

**Figure 6:**
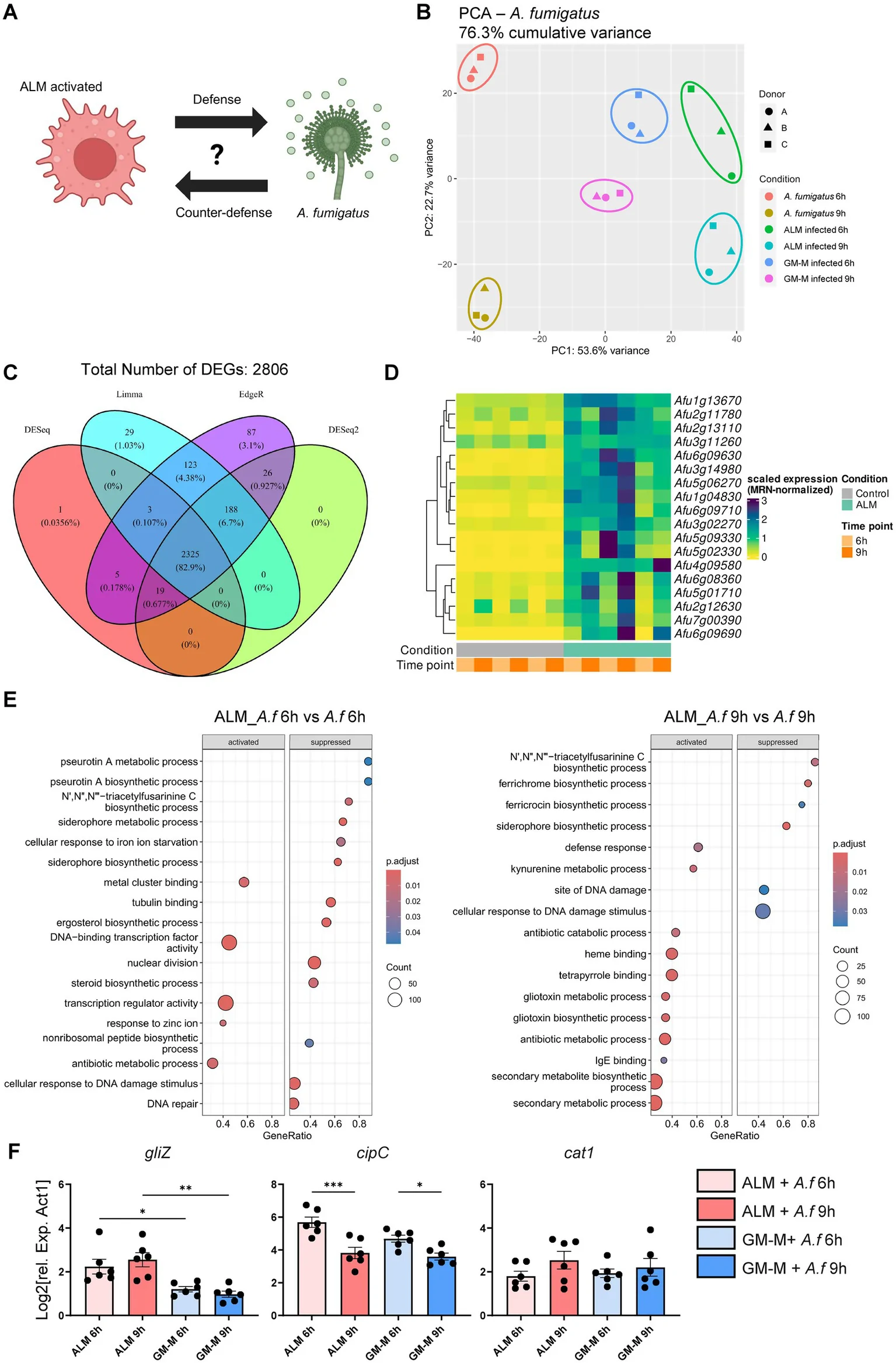
Analysis of the fungal transcriptome reveals a strong counter-defense response of *A. fumigatus* upon ALM stimulation used for immune evasion. **(A)** Graphical illustration to visualize the idea behind the dual RNA-sequencing analysis to compare both human and fungal transcriptomic profiles. Created with Biorender.com. **(B)** PCA plot of the dual RNA-sequencing analysis visualizing differences in the *A. fumigatus* transcriptomic profile. **(C)** Venn diagram of fungal DEGs after 9 h of challenge with ALM. **(D)** Heatmap of respective virulence genes expressed by *A. fumigatus* in contact to ALM next to the respective unchallenged control. **(E)** Representative pathways regulated by *A. fumigatus* transcriptome after 6 h and 9 h of ALM encounter. The dot plot visualizes pathways activated or suppressed by *A. fumigatus* ATCC46645 upon ALM challenge. **(F)** Validation of selected genes upregulated in *A. fumigatus* upon Mo challenge. *n* = 6 +/− SEM, Ordinary One-Way ANOVA, Šidák’s Multiple Comparison; * *p* < 0.05, ** *p* < 0.01, *** *p* < 0.001.

During interaction with GM-Ms, *A. fumigatus* also adjusted its transcriptional program but to a lesser extent. At 9 h, fewer DEGs were detected (1,331 DEGs; Supplementary Figure S6B), and induction of virulence-associated genes was comparatively reduced (Supplementary Figure S6A). While suppression of growth-related pathways was also evident at 6 h (Supplementary Figure S6C), GM-M interaction preferentially induced pathways associated with biofilm formation, which were not prominently activated during ALM interaction. Notably, key pathways such as gliotoxin biosynthesis and kynurenine metabolism were not significantly induced in response to GM-Ms. We validated these dual RNA-sequencing results by qRT-PCR analysis of selected fungal genes (*gliZ*, *cat1*, and *cipC*), confirming the observed transcriptomic profiles at both time points (Figure 6F).

Together, these results indicate that *A. fumigatus* mounts a more pronounced and coordinated counter-defense response during interaction with ALMs. This enhanced host-pathogen interplay not only reflects the stronger immune activation elicited by ALMs but also facilitates the identification of fungal adaptation mechanisms, including the induction of genes such as *aspf1*, that may be less apparent in alternative Mφ models such as GM-Ms.

### 3.5 Host-pathogen interaction networks highlight coordinated ALM and *A. fumigatus* infection responses

To further characterize the bidirectional interaction between ALMs and *A. fumigatus*, we constructed predicted host-pathogen protein–protein interaction (PPI) networks based on the dual transcriptomic dataset as described in the methods section. Therefore, we prioritized candidate proteins based on features associated with host-pathogen interactions, including predicted GPI-anchors, transmembrane domains, signaling peptides, secretion signals and subcellular localization. PPI networks were computed using STRING DB. Consistent with the pathway enrichment analyses above, network analysis revealed a persistent modulation of *A. fumigatus* interaction on the host aerobic electron transport chain-associated proteins at both investigated time points (Figures 7A,B). In parallel, ALMs robustly modulated a central inflammatory network enriched for TNF signaling-associated proteins, including ICAM1, TLR2, CSF1, LILRB1, and CXCL10. Employing the STRING DB enrichment module, we found that ALMs exhibited a cluster visualizing a pronounced CXCL10-associated inflammatory signature at 6 h post-infection, whereas expression of both CSF2 receptor subunits became more prominent at 9 h, indicating sustained immune activation and Mφ responsiveness. On the fungal side, the predicted interaction landscape initially emphasized proteins associated with galactose metabolism and protein translation at 6 h post-infection. By 9 h, however, the fungal network shifted toward a pronounced counter-defense response signature characterized by proteins linked to stress adaptation, respirasome-associated proteins, and responses to reactive oxygen species, suggesting adaptation to increasing host-imposed immune pressure. Notably, the analysis revealed several established virulence-associated proteins, including the antioxidant enzymes catalase Cat1 and superoxide dismutase SodB. Collectively, these host-pathogen interaction networks further support the concept that ALMs mount a coordinated inflammatory response that induces extensive fungal counteradaptation during early infection.

**Figure 7:**
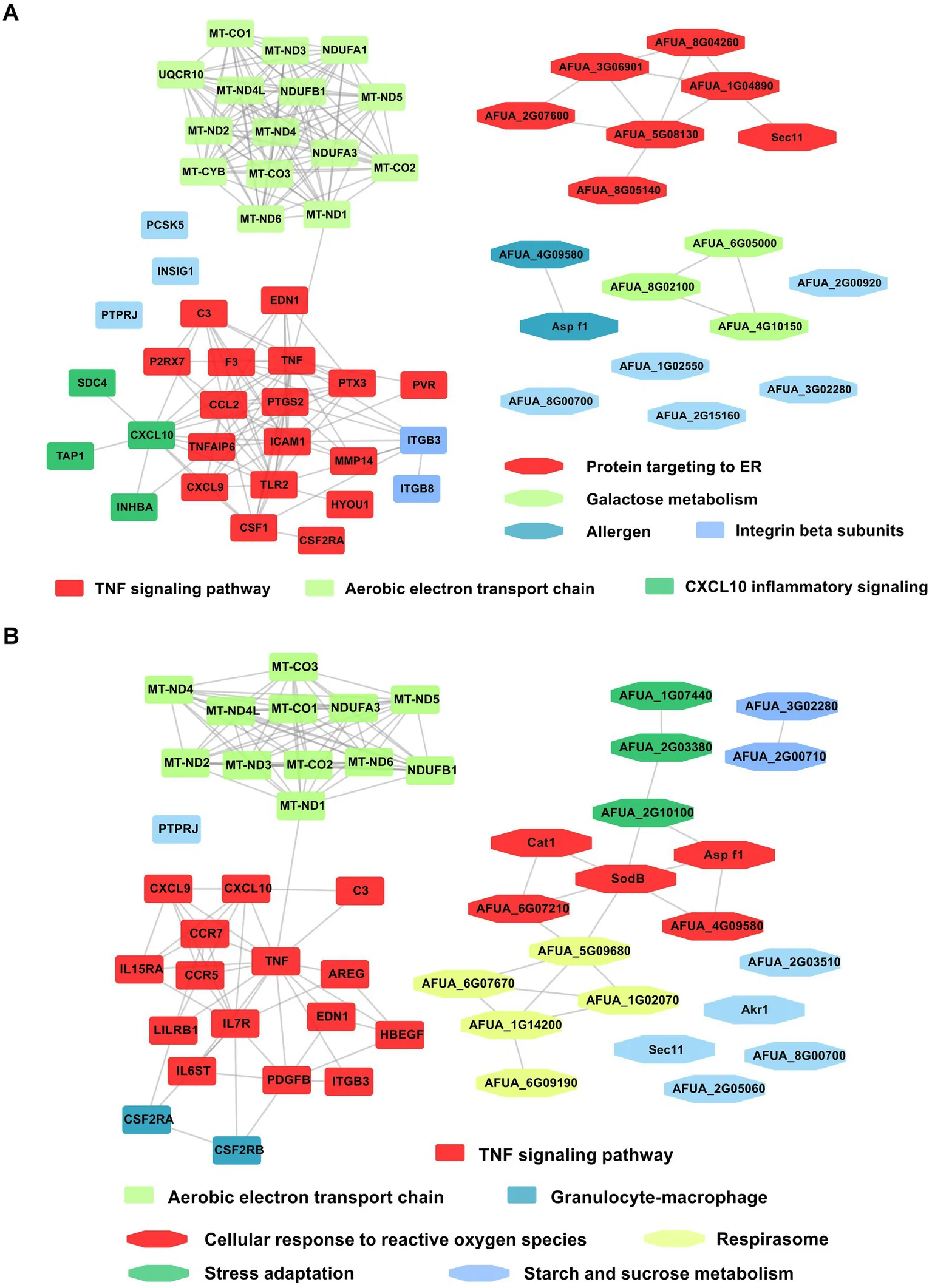
Host-pathogen protein–protein interaction (PPI) networks generated from the dual RNA-sequencing dataset. The plots display the PPIs **(A)** 6 h and **(B)** 9 h. The networks were constructed as indicated in the methods section. Modules and interactions were identified using STRING DB. Node shapes distinguish host proteins from pathogen-associated proteins, whereas edges indicate interactions between or functional influence on the proteins.

### 3.6 Fungal allergens contribute to immune evasion during macrophage interaction

The induction of allergen-encoding genes *aspf1* and *aspf2* in our dual RNA-sequencing analysis prompted us to investigate their functional role during early Mφ interactions. We infected pAMs, ALMs, and GM-Ms with *A. fumigatus* wild type (WT; strain CEA17 ∆*akuB KU80*) and the corresponding knockout mutant strains ∆*aspf1* and *∆aspf2* for 9 h. Subsequently, we quantified cytokine and chemokine secretion using cytometric bead array (CBA) analysis.

Consistent with our previous observations, pAMs and ALMs mounted robust cytokine responses upon infection, whereas GM-Ms showed limited responsiveness relative to uninfected controls (Figure 8A). Elevated baseline cytokine levels in GM-M, including CCL2, CCL3, CXCL10, and IL-8 further support their pre-activated phenotype. Comparative analysis of WT and mutant strains revealed largely comparable cytokine responses across all three Mφ populations. However, infection with the Asp f1-deletion mutant resulted in enhanced cytokine induction relative to uninfected controls for several mediator including IL-1b, IL-10, CCL4, CCL8, CXCL10, TNF, IL-6, or IL-8, depending on the Mφ type (Figures 8A,B; Supplementary Figure S7A). Notably, GM-Ms displayed significant induction of TNF and CXCL10 only in response to the Asp f1-deficient strain, but not to WT infection, suggesting that Asp f1 may modulate host inflammatory responses.

**Figure 8:**
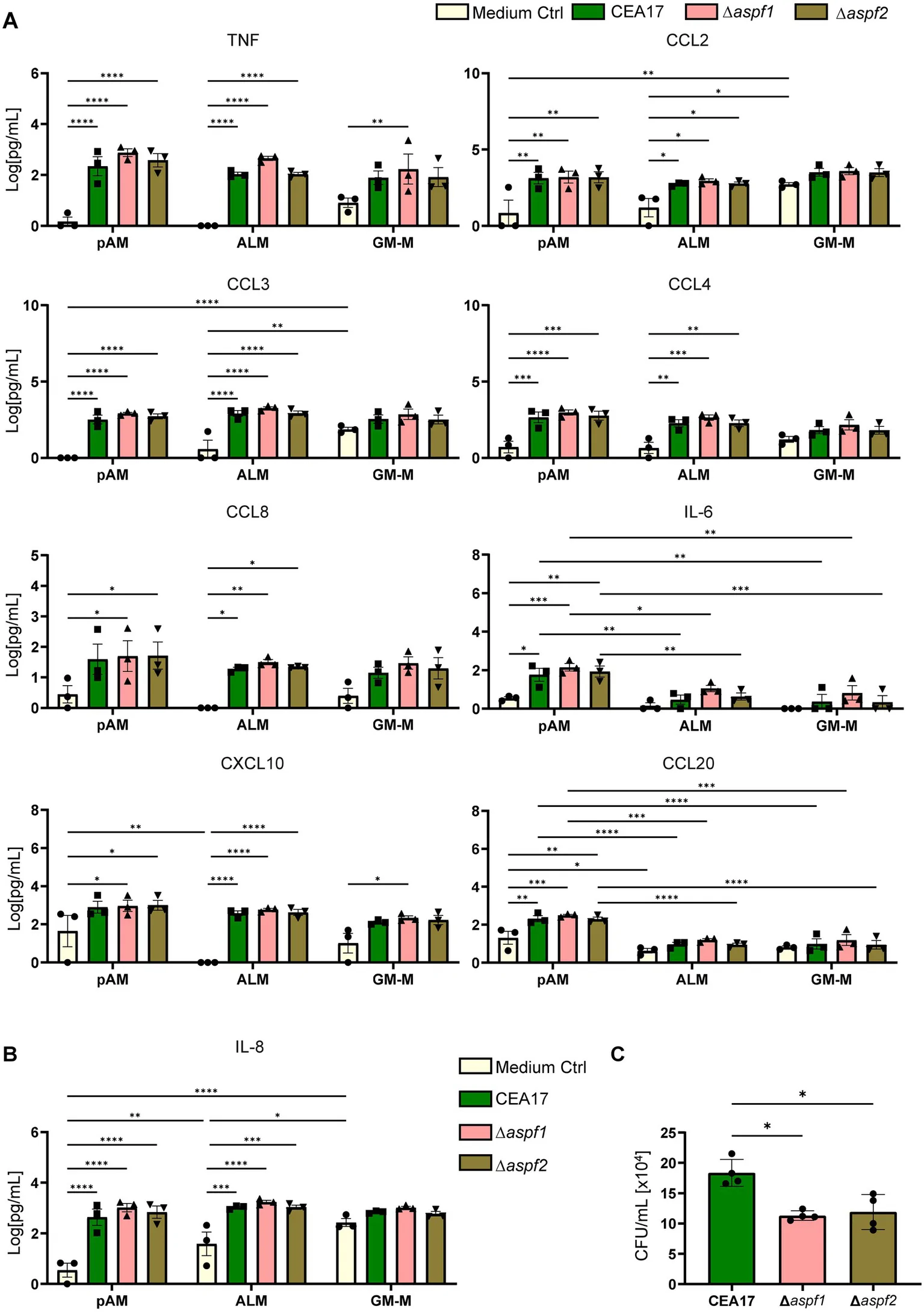
pAM and ALM show strong infection responses upon challenge with *A. fumigatus* CEA17, Aaspf1 and Aaspf2. **(A)** Cytokine secretion analysis of pAM, ALM and GM-M infected with MOI 5 of *A. fumigatus* CEA17 or its respective knockout mutants after 9 h. Cytokines were analyzed using a custom LEGENDplex CBA kit. The values were transformed using log(Y + 1). *n* = 3 +/− SEM; Two-Way ANOVA, Tukey’s Multiple Comparison; * *p* < 0.05, ** *p* < 0.01, *** *p* < 0.001, **** *p* < 0.0001. **(B)** Singleplex ELISA analysis of IL-8 secreted by pAM, ALM or GM-M upon *A. fumigatus* CEA17, *Δaspf1* and *Δaspf2* MOI 5 infection. *n* = 3 +/− SEM; Two-Way ANOVA, Tukey’s Multiple Comparison; * *p* < 0.05, ** *p* < 0.01, *** *p* < 0.001, **** *p* < 0.0001. **(C)** Colony forming unit analysis of ALMs infected with MOI 5 of *A. fumigatus* CEA17 or its respective knockout mutants after 9 h. ALMs were lysed with cold ddH2O and the conidia suspension was plated on malt agar plates at 37C for 18 h. *n* = 4 +/− SEM, RM-ANOVA, Dunnett’s Multiple comparison; * *p* < 0.05.

To further assess the functional impact of allergen deficiency, we quantified fungal survival following infection of ALMs using colony-forming unit (CFU) assays. Both ∆*aspf1* and *∆aspf2* showed significantly reduced CFU recovery compared to WT (Figure 8C), indicating enhanced Mφ-mediated fungal killing by ALMs in the absence of these allergens. To exclude confounding effects on fungal germination or uptake, control experiments confirmed that these differences were not attributable to altered fungal growth or phagocytic uptake (Supplementary Figures S7B–D). Together, these findings indicate that the allergens Asp f1 and Asp f2 contribute to fungal persistence during Mφ interaction, at least in part by contributing to immune evasion. Their absence enhances Mφ-mediated killing in ALMs, and modestly augments cytokine responses, emphasizing their role in shaping host-pathogen dynamics.

## 4 Discussion

Invasive fungal infections including IPA remain a major cause of morbidity and mortality in immunocompromised patients, particularly when diagnosis is delayed or insufficiently sensitive (Elalouf et al., 2023; Kousha et al., 2011; Wichmann et al., 2025). The environmental mold *A. fumigatus* deploys multiple highly specialized immune evasion strategies, including masking of pathogen-associated molecular patterns by melanin and the hydrophobin RodA, resistance to oxidative stress, and rapid germination at physiological temperatures of the host (Liu et al., 2024; Heinekamp et al., 2015). Upon inhalation, pAMs serve as the first line of defense by encountering inhaled conidia, thereby maintaining pulmonary homeostasis and initiating antifungal immunity (Balloy and Chignard, 2009; Yan et al., 2025). However, the early cellular and molecular determinants impacting fungal clearance versus progression to invasive disease remain insufficiently understood, in part due to the lack of physiologically relevant *in vitro* models.

In the present study, we adapted and systematically validated a previously established ALM model derived from human primary monocytes and demonstrate that these cells closely recapitulate key phenotypic and functional features of pAMs. Consistent with previous work (Pahari et al., 2023), ALMs exhibited a round morphology and expressed canonical Mφ markers, closely resembling pAMs, whereas GM-Ms displayed a divergent phenotype. Notably, the expression of MHC II molecules and the scavenger receptor CD68, key mediators of antigen presentation and phagocytic activity (Chen and Jensen, 2008; Chistiakov et al., 2017; Zhang et al., 2022), aligned closely between ALMs and pAMs, while GM-Ms differed across all assessed markers. Functionally, ALMs mirrored pAM-like cytokine responses upon *A. fumigatus* challenge, independent of fungal load and infection time. Specifically, TNF, CCL3, IL-1b, and IL-8, hallmark mediators of antifungal immunity (Shankar et al., 2024) were significantly induced in ALM and pAM, whereas GM-M exhibited elevated baseline cytokine levels, indicative of a pre-activated state. These findings demonstrate that ALMs maintain a homeostatic steady-state under resting conditions while mounting a rapid inducible response upon pathogen challenge, characteristic of tissue-resident Mφs (Mosser et al., 2021; Sun et al., 2026). In contrast, GM-Ms appear constitutively primed, likely due to GM-CSF-driven differentiation (Lotfi et al., 2020; Petrina et al., 2024). Together, these results highlight how differentiation context shapes Mφ identity and emphasize the limitations of the employed conventional GM-CSF-based model. Using the FLARE-based assay, we directly quantified phagocytosis and killing of *A. fumigatus,* two critical early defense mechanisms against inhaled fungal conidia (Luther et al., 2008; Jhingran et al., 2012). ALMs internalized conidia more rapidly and efficiently than GM-Ms across all tested conditions. This behavior reflects the physiological role of alveolar Mφs, which continuously survey the airway environment and rapidly clear particulate matter (Luther et al., 2008; Neupane et al., 2020). Although, intracellular killing efficacy rates were broadly comparable, the accelerated uptake kinetics of ALMs likely provide a decisive advantage by restricting fungal germination, a key step in disease progression (Savers et al., 2016). Notably, ALMs sustained phagocytic activity under high fungal loads and increased killing over time, underscoring their functional adaptability.

At the transcriptional level, paired donor analyses further strengthened the pAM resemblance of ALMs and revealed that ALMs undergo rapid and coordinated activation upon fungal challenge. Infection triggered metabolic reprogramming was characterized by downregulation of oxidative phosphorylation, the predominant energy-generating pathway in tissue-resident alveolar Mφs (Woods and Mutlu, 2025) and consistent with established Mφ activation programs during infection (Wculek et al., 2023; Hatinguais et al., 2021; Gonçalves et al., 2020). Concurrently, ALMs strongly induced pro-inflammatory transcriptional programs, including TNF- and NF-kB-signaling pathways, alongside chemokines such as CCL3 and CXCL10 that drive immune cell recruitment (Shankar et al., 2024). These results are consistent with the established role of inflammatory signaling in antifungal host defense (Mezger et al., 2008; Guo et al., 2020). In contrast, GM-Ms responded more slowly and less synchronously, reinforcing the concept that Mφ differentiation state dictates both the timing and magnitude of antifungal immunity. While public datasets of healthy pAMs provide an appropriate reference for validating the basal alveolar identity of ALMs, comparable transcriptome-wide datasets from *A. fumigatus*-stimulated human pAMs are currently lacking. Consequently, our study provides one of the first comprehensive characterizations of early human alveolar Mφ-like transcriptional response to this clinically relevant fungal pathogen.

An intriguing result of this study is the enhanced and prolonged uptake of *A. fumigatus* conidia by ALMs, which closely resembles the phagocytic behavior of pAMs. Consistent with prior studies (Ibrahim-Granet et al., 2003; Philippe et al., 2003; Shevchenko et al., 2013) we observed a time-dependent increase in phagocytic efficacy by ALMs especially at higher MOIs.

Although the molecular mechanisms underlying this phenotype were beyond the scope of this present study, our transcriptomic data suggests that differences in pathogen recognition and intracellular processing may contribute to the distinct functional properties of ALMs. We observed surface expressions of the pathogen-recognition receptors (PRRs) TLR2 and Dectin-1 on ALMs and GM-Ms (Supplementary Figure S3D) both of which are described to play central roles in *A. fumigatus* phagocytosis (Goodridge et al., 2011; Luther et al., 2007).

Dectin-1, a C-type-lectin receptor on innate immune cells such as Mφs of both human and mice, recognizes β-glucan structures on swollen and germinated conidia (Margalit and Kavanagh, 2015), thereby activating phagocytosis (Goodridge et al., 2011). Further, inhibition of Dectin-1-ligand binding with a blocking antibody or laminarin was associated with reduced phagocytosis of *A. fumigatus* conidia. Similarly, TLR2 deficiency or antibody blocking reduced conidial phagocytosis (Luther et al., 2007). Therefore, we investigated whether ALMs and GM-Ms show differences in Dectin-1 and TLR2 surface expression, which we did not observe. In contrast to published data on tissue-resident pAMs exposed to the alveolar environment (Taylor et al., 2002; Steele et al., 2005), ALM and GM-M models establish a low but defined basal level of PRR expression in the absence of exogenous niche priming. This suggests that other factors may contribute to the observed distinctions in phagocytic efficacy. Still, these PRRs, together with pathways regulating phagosome maturation and oxidative antimicrobial responses, represent promising candidates for future mechanistic studies of ALMs. In addition, due to the central role of pAM in the continuous clearance of inhaled fungal conidia, these mechanisms for fungal uptake and host defense may also be relevant in conditions associated with fungal persistence or chronic pulmonary fungal exposure, including allergic bronchopulmonary aspergillosis (Jones et al., 2021; Kowalski et al., 2016).

In our previous study (Zoran et al., 2019), we identified etanercept treatment and reduced monocyte counts as risk factors for IPA in a clinical setting and demonstrated that etanercept can impair Mφ-associated immune responses to *A. fumigatus*. Specifically, modulation of the TNF- and CXCL10-associated responses, as well as effects on NF-kB-related signaling pathways, suggested that disruption of key inflammatory circuits may compromise antifungal immunity. Based on these results, the present study provides mechanistic support that ALMs rapidly activate these critical pathways upon infection. In contrast, the delayed response observed in GM-Ms suggests reduced responsiveness to pathogen-specific cues. Mechanistically, the rapid and coordinated activation in ALMs likely reflects enhanced pathogen sensing and efficient signal transduction, enabling effective early immune control despite fungal immune evasion strategies.

Recently, a murine *in vitro* model of fetal-liver derived alveolar-like macrophages (FLAMs) was published in which fetal-liver derived cells are differentiated in culture medium supplemented with both GM-CSF and TGFβ (Gorki et al., 2022; Thomas et al., 2022; Thomas et al., 2026). Ammar and Olive (2026) systematically refined and evaluated this model as a robust and experimentally tractable system resembling murine primary alveolar Mφs to investigate pulmonary Mφ biology. Across a series of studies, they demonstrated that the combination of GM-CSF and TGFβ during differentiation recapitulates key phenotypic, metabolic and transcriptional features of murine resident alveolar Mφs and, that both cellular origin and alveolar differentiation fundamentally shape Mφ responses to pulmonary stimuli and pathogens (Gilliland et al., 2026; Wiggins et al., 2025; Ammar and Olive, 2026).

Our study supports and extends this concept in a human monocyte-derived alveolar-like Mφ model. In coherence with the observations of Ammar and Olive (2026), we show that alveolar differentiation is associated with distinct transcriptional and functional patterns compared with conventionally differentiated Mφs. Direct comparison of human ALMs with pAMs and GM-Ms demonstrated that this alveolar differentiation state influences key phenotypic and early inflammatory response to *A. fumigatus*, resulting in distinct transcriptional programs. This pattern includes ALM specific CXCL10- and interferon-associated signaling, which was analogously observed in a previous study of the FLAMs model stimulated with TLR2 agonists including fungal zymosan (Thomas et al., 2026), especially in the FLAMs cultures with TGFβ.

While the FLAMs model was established as a versatile platform for investigating pulmonary host-pathogen interactions including *Cryptococcus neoformans* (Wiggins et al., 2025), the primary causative agent of pulmonary cryptococcosis, our study extends this concept to the clinically relevant filamentous fungus *A. fumigatus*. This study complements the murine FLAM model by translating the adapted alveolar-like Mφ concept to a human monocyte-derived system, thereby enabling the investigation of human-specific antifungal immune responses. Together, these findings reinforce the concept that alveolar differentiation is not merely a phenotypic feature but a major determinant of pathogen-specific innate immune programming and underscore the value of the alveolar-like Mφ models for mechanistic studies of human respiratory host-pathogen interactions.

While our past work (Zoran et al., 2019) focused primarily on human signaling responses, by integrating dual RNA-sequencing in our current study, we captured the dynamic interplay between host defense and fungal adaptation. Particularly, *A. fumigatus* mounted a strikingly stronger transcriptional counter-response when challenged with ALMs, including the induction of cytotoxic secondary metabolites like gliotoxin (Sun et al., 2026) or oxidative stress defenses such as *cat1* (Abad et al., 2010; Keizer et al., 2022). This implies a tightly coupled interaction in which enhanced host immune activation directly triggers fungal adaptation mechanisms. Such bidirectional regulation was not accessible in our previous study and highlights the added value of the ALM model combined with dual transcriptomics. Markedly, the substantially stronger transcriptional response of *A. fumigatus* challenged with ALMs compared to GM-Ms included upregulation of oxidative stress defense genes such as *cat1* (Keizer et al., 2022; Abad et al., 2010), as well as genes involved in gliotoxin biosynthesis. Gliotoxin is a major virulence factor known to impair host immune function by inducing apoptosis, inhibiting phagocytosis, and suppressing NF-kB-signaling (Günther et al., 2024; Knowles et al., 2020). Furthermore, we found an upregulation of *A. fumigatus* kynurenine metabolism, a potential immune evasion mechanism to dampen the host inflammatory response (Gargaro et al., 2016; Zelante et al., 2021). The enhanced induction of gliotoxin and kynurenine biosynthesis pathways in response to ALMs indicates that the fungus may perceive these cells as a more potent immune threat, thereby activating more robust counter-defense strategies.

Analysis of the predicted PPI network further emphasizes the profound impact of *A. fumigatus* on ALM responses. The identified interaction clusters reinforced our pathway enrichment analyses and provided additional evidence that fungal challenge profoundly affects host oxidative phosphorylation, a process increasingly recognized as a central component of Mφ adaptation during fungal infection (Hatinguais et al., 2021; Gonçalves et al., 2020). In parallel, the host network prominently featured TNF-associated signaling modules, including ICAM1, TLR2, CSF1 and CXCL10, further supporting the central role of inflammatory signaling during antifungal defense and aligning with our previous work (Zoran et al., 2019). Experimental studies previously demonstrated that TNF pre-treatment enhances resistance to *A. fumigatus* in infection in neutropenic mice by increasing Mφ phagocytic activity, whereas TNF-neutralization results in elevated fungal burden and mortality in IPA mouse models (Balloy and Chignard, 2009). Consistent with these observations, TLR2-mediated recognition of both conidia and hyphae induces TNF expression, while loss of TLR2 markedly impairs this response (Mambula et al., 2002; Balloy and Chignard, 2009). Moreover, our previous work linked TNF-associated signaling to CXCL10 induction during *A. fumigatus* infection (Zoran et al., 2019), a relationship that was recapitulated in the current PPI network analysis. Together, these findings further support an important role of the TNF-CXCL10 axis in orchestrating early fungal immunity.

On the fungal side, the predicted interaction networks identified a distinct cluster of oxidative stress response factors, including the antioxidant enzymes Cat1 and SodB, closely associated with proteins involved in the respirasome and energy metabolism (Wu et al., 2020). During host-pathogen interaction, reactive oxygen and nitrogen species generated by Mφs impair fungal respiration and mitochondrial function (Joseph-Horne et al., 2001), necessitating efficient detoxification mechanisms. In this context, SodB expression appears to fulfill a central protective role and has additionally been implicated in energy metabolism during early fungal germination in *A. nidulans*. Consistent with these functions, deletion of *sodB* increases susceptibility to Mφ-mediated killing (Pákozdi et al., 2024; Kanamaru et al., 2025). The coordinated induction of oxidative stress defense pathways together with respiratory adaptation mechanisms therefore suggests that *A. fumigatus* actively remodels its metabolism to withstand ALM-imposed immune pressure.

Beyond classical virulence factors stated above, we identified a significant induction of allergen-encoding genes, including *aspf1* and *aspf2* as functionally relevant mediators of host-pathogen interaction upon ALM challenge. Deletion of these genes reduced fungal survival and increased susceptibility to ALM-mediated killing. Our results suggest that fungal allergens may contribute to immune evasion beyond their established role in allergic sensitization. One potential explanation is that these proteins modulate host immune signaling or interfere with intracellular killing mechanisms, thereby promoting fungal persistence. Mitogillin, encoded by *aspf1*, is a ribotoxin contributing to *A. fumigatus* pathogenicity (Liu et al., 2018) that cleaves a phosphodiester bond in the 28S ribosomal RNA, thereby inhibiting protein synthesis and inducing apoptosis (Morton et al., 2011; Lacadena et al., 2007). On the other hand, Asp f2 was identified as a central immune evasion and complement regulating protein with the ability to bind to Factor H, factor-H-like proteins and plasminogen. Hence, blocking the host innate immune response and disrupting lung epithelial cell layers to assist in early fungal infection and tissue penetration (Dasari et al., 2018). Suitable to the described role of Asp f1 as a protein biosynthesis inhibitor (Lacadena et al., 2007), our observation that ∆*aspf1* conidia induced enhanced cytokine response in our setup extend these functions by demonstrating these allergens actively modulate Mφ responses during early infection. Importantly, these fungal adaptations were substantially attenuated during interaction with GM-Ms, where fewer virulence-associated pathways were induced. This finding underscores that the choice of Mφ model not only shapes host responses but also fundamentally influences pathogen behavior, potentially masking critical virulence mechanisms in less representative systems.

One potential influencing factor to consider regarding the transcriptomic profile, is the impact of growth and storage conditions on the phenotypic and transcriptomic profile of filamentous fungi. Many studies have discussed such influential factors including humidity, brightness, oxygen saturation and storage period (Zain et al., 2009; Wang et al., 2021; Kang et al., 2021; Lamarre et al., 2008). Malt agar is frequently recommended for aerobiological studies and provides optimal growth conditions for *A. fumigatus* (Viegas et al., 2021; Zain et al., 2009). Therefore, we sporulated all *A. fumigatus* strains on malt agar used for infection experiments except the FLARE assay, where we ensured stable reporter expression by sporulation on AMM plates as described in the original publication. Still, the physical and chemical factors of chosen nutrient types as well as the growth environment can exhibit a priming role on infection relevant components like conidia morphology, secondary metabolites, and virulence genes (Kang et al., 2021; Wang et al., 2021). A published study has discussed that varying plate incubation conditions including temperature shocks, brightness as well as initial removal from the conidiophore changes the conidia transcriptomic profile (Wang et al., 2021). While conidia stored in the conidiophore still show transcriptional activity and are responsive to their environment, dormancy is established after conidial detachment from the conidiophore. In contrast to conidia attached to the conidiophore, detached conidia showed suppression of the developmental regulator WetA as well as unchanged transcriptional responses upon temperature shocks with missing heat shock protein expression (Wang et al., 2021).

The choice of malt agar might have an influence on the transcriptomic profile observed in our dual RNA-Sequencing including protein expression of gliotoxin and Asp f1 during infection. Still, the specific significant increase of those virulence factors upon ALM encounter suggests that the choice of Mφ population and differentiation exerts a crucial role on the fungal counter-defense strategy as well. Moreover, each ALM or GM-M donor was infected with the same stock of *A. fumigatus* conidia to ensure consistency across Mφ populations within a given donor. Conclusively, the selection of growth medium and environment can have an impact on *A. fumigatus* phenotypic and transcriptomic profile during infection response as well as the choice of immune cell populations and differentiation conditions, as we observed in this study.

Despite the above discussed advances, several limitations of our study should be considered. First, while our method of cell isolation from lung biopsies yields a highly enriched pAM population (~91%) defined by typical pAM markers CD11c^+^/CD206^+^ double positivity (Schyns et al., 2018; Bain and MacDonald, 2022; Bharat et al., 2016) as well as high autofluorescence (Aegerter et al., 2022), contamination with a small number of interstitial Mφs (IMs) cannot be fully excluded. Additionally, we performed mechanical disruption without further enzymatic digestion, typically employed to isolate IMs from tissue (Jeong and Seok, 2025; Atif et al., 2018; Aegerter et al., 2022) to further minimize the occurrence of IMs in the remaining cell population. Second, it is described that pAMs can lose their phenotype quickly in *ex vivo* culture systems (Papp et al., 2018). To minimize this event, pAMs were experimentally evaluated within 48 h of tissue removal. Still, slight phenotypic drifts might occur in this time span and must be considered an important limitation in this study. While published literature discusses that supplementation of GM-CSF to the pAM culture medium does not lead to changes or loss of the pAM phenotype in prolonged *in vitro* culture (David et al., 2025), the impact of the ALM culture components on pAMs has to be elucidated. Similarly, the ALM phenotype depends on defined differentiation conditions and may exhibit temporal variability, particularly after withdrawal of key factors (Pahari et al., 2023). Although ALMs closely resemble pAMs phenotypically as well as mechanistically, they remain an *in vitro* generated model and therefore cannot fully recapitulate the developmental origin, life-long tissue adaptation and complex microenvironmental imprinting of tissue-resident pAMs (Bain and MacDonald, 2022). Further, our experimental system relies on monocultures and therefore does not capture the full complexity of the alveolar niche, including epithelial interactions and multicellular immune networks. Incorporation of co-culture systems or organoid models will be essential to validate and extend these findings. Additionally, our computational approach did not identify direct host-pathogen protein interactions. Future studies could improve these analyses by integrating additional bioinformatic resources and interaction databases, including ortholog- and homology-based datasets derived from other fungal species like *Saccharomyces cerevisiae* or *Candida albicans* (Remmele et al., 2015). Integration of these resources may help to refine predicted interaction networks and advance the mechanistic understanding of alveolar Mφ-*A. fumigatus* interactions during early infection. Lastly, while key phenotypic and functional characteristics were experimentally validated, comprehensive mechanistic validation of all differentially expressed pathways identified by transcriptomic analyses was beyond the scope of this study. Likewise, predicted host-pathogen interaction networks are based on computational inference. Hence, they should be regarded as hypothesis-generating rather than experimentally validated molecular interactions. In addition, the current ALM model reflects the biology of healthy donor-derived Mφs rather than the altered immune landscape of immunocompromised patients at high risk for IPA (Hohl, 2017). Nevertheless, the close phenotypic, functional and transcriptomic similarities of ALMs to pAMs, together with their experimental accessibility and reproducibility, establishes this model as a valuable platform for mechanistic studies of human antifungal host-pathogen interactions. Moreover, the transcriptomic resource and predicted interaction networks generated in the present study provide a valuable framework for future targeted functional validation and for the continued refinement of human pAM models, including the incorporation of patient-derived cells and additional components of the alveolar niche.

Collectively, our study demonstrates that ALMs provide a robust and physiologically relevant, experimentally accessible *in vitro* platform for investigating early interactions in pulmonary aspergillosis (Figure 9). Compared to GM-Ms, ALMs more accurately reproduce the steady-state phenotype, rapid immune activation kinetics, and functional responses of pAMs. By enabling simultaneous analysis of host and pathogen programs, this model provides new insights into the dynamic balance between immune defense and fungal counter-defense. Beyond aspergillosis, ALMs offer a versatile platform for studying a broad range of respiratory infections and for identifying therapeutic targets that enhance host immunity.

**Figure 9:**
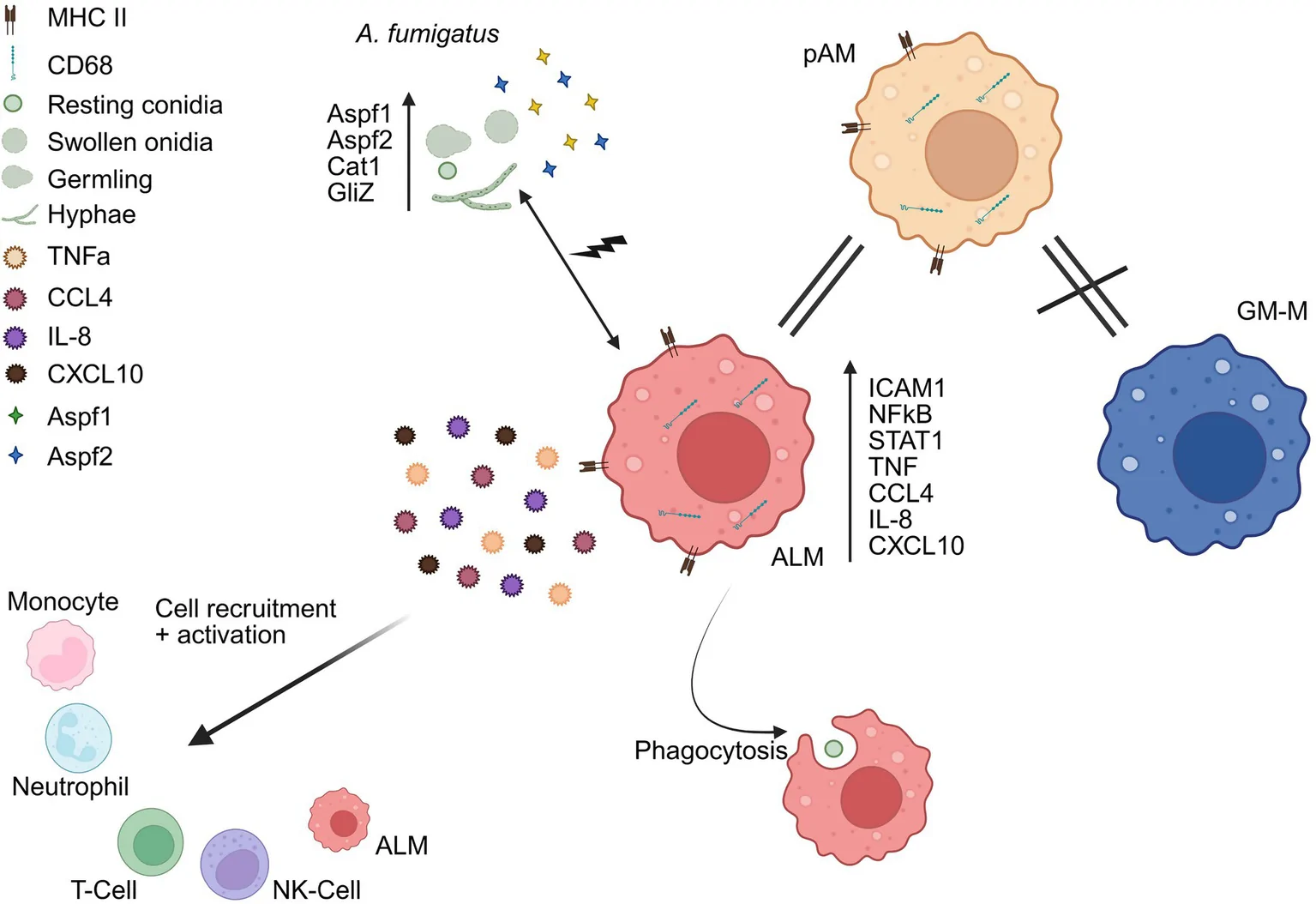
ALM are an efficient pAM resembling *in vitro* model suitable to study *A. fumigatus* infection. Summary displaying the results found in the research study. ALM resemble a pAM phenotype in steady-state as well as during infection, which we did not find for GM-M. Upon *A. fumigatus* infection, ALMs rapidly phagocytose *A. fumigatus* conidia as well as upregulate pro-inflammatory genes including cytokines and chemokines, to attract other immune cell types like neutrophils and monocytes to eliminate the fungus. On the fungal side, *A. fumigatus* encounters ALM as an important immune threat and upregulates genes encoding immune evasion mechanisms including gliotoxin and the allergens Asp f1 and Asp f2 for fungal survival. Created with Biorender.com.

## Data Availability

The datasets presented in this study can be found in online repositories. The names of the repository and accession number can be found below: http://www.ebi.ac.uk/arrayexpress and accession number E-MTAB-17130.
